# Agonist-induced membrane nanodomain clustering drives GLP-1 receptor responses in pancreatic beta cells

**DOI:** 10.1371/journal.pbio.3000097

**Published:** 2019-08-20

**Authors:** Teresa Buenaventura, Stavroula Bitsi, William E. Laughlin, Thomas Burgoyne, Zekun Lyu, Affiong I. Oqua, Hannah Norman, Emma R. McGlone, Andrey S. Klymchenko, Ivan R. Corrêa, Abigail Walker, Asuka Inoue, Aylin Hanyaloglu, Jak Grimes, Zsombor Koszegi, Davide Calebiro, Guy A. Rutter, Stephen R. Bloom, Ben Jones, Alejandra Tomas

**Affiliations:** 1 Section of Cell Biology and Functional Genomics, Division of Diabetes, Endocrinology and Metabolism, Department of Medicine, Imperial College London, London, United Kingdom; 2 Department of Cell Biology, Institute of Ophthalmology, University College London, London, United Kingdom; 3 Section of Investigative Medicine, Division of Diabetes, Endocrinology and Metabolism, Department of Medicine, Imperial College London, London, United Kingdom; 4 Laboratoire de Bioimagerie et Pathologies, UMR CNRS 7021, University of Strasbourg, Illkirch-Strasbourg, France; 5 New England Biolabs, Ipswich, Massachusetts, United States of America; 6 Department of Surgery and Cancer, Imperial College London, London, United Kingdom; 7 Tohoku University, Sendai, Japan; 8 Institute of Metabolism and Systems Research, University of Birmingham, Birmingham, United Kingdom; 9 Centre of Membrane Proteins and Receptors (COMPARE), Universities of Birmingham and Nottingham, Birmingham and Nottingham, United Kingdom; University of Pennsylvania, UNITED STATES

## Abstract

The glucagon-like peptide-1 receptor (GLP-1R), a key pharmacological target in type 2 diabetes (T2D) and obesity, undergoes rapid endocytosis after stimulation by endogenous and therapeutic agonists. We have previously highlighted the relevance of this process in fine-tuning GLP-1R responses in pancreatic beta cells to control insulin secretion. In the present study, we demonstrate an important role for the translocation of active GLP-1Rs into liquid-ordered plasma membrane nanodomains, which act as hotspots for optimal coordination of intracellular signaling and clathrin-mediated endocytosis. This process is dynamically regulated by agonist binding through palmitoylation of the GLP-1R at its carboxyl-terminal tail. Biased GLP-1R agonists and small molecule allosteric modulation both influence GLP-1R palmitoylation, clustering, nanodomain signaling, and internalization. Downstream effects on insulin secretion from pancreatic beta cells indicate that these processes are relevant to GLP-1R physiological actions and might be therapeutically targetable.

## Abstract 2

Nanodomain segregation and clustering of the glucagon-like peptide-1 receptor, a key target for type 2 diabetes therapy, is regulated by agonist binding, leading to compartmentalization of downstream signaling and clathrin-dependent internalization and impacting pancreatic beta cell responses.

## Introduction

G protein–coupled receptors (GPCRs), the largest membrane receptor family in eukaryotes [1], are integral membrane proteins, and, as such, both their physical organization and their signaling properties are modulated by the lipid composition of the surrounding membrane [2,3]. The localization of GPCRs to dynamic membrane nanodomains has been widely reported [4–6]. These nanodomains, or membrane rafts, which cannot be directly observed in living cells with current methods [7], are often described as highly organized detergent-resistant, liquid-ordered, glycosphingolipid- and cholesterol-rich platforms where receptor-signaling complexes become compartmentalized, facilitating efficient coupling with G proteins [5,8,9]. Additionally, most GPCRs are modified posttranslationally with one or more palmitic acid chains linked covalently, but reversibly, via a thioester bond to cysteines within the intracellular domain of the receptor, in a process known as palmitoylation [2,10]. The insertion of acyl chain(s) is regulated by families of acyltransferases (DHHCs) and palmitoyl protein thioesterases [11,12] and can be either constitutive or modulated by agonist binding [13,14]. GPCR palmitoylation at or near the end of its C-terminal tail creates a new membrane anchor and a further intracellular loop [15] that modifies the GPCR structure and its interactions with specific intracellular partners, favoring receptor partitioning into membrane nanodomains [2,16,17].

Class B GPCRs, such as the glucagon-like peptide-1 receptor (GLP-1R), play crucial roles in the control of glucose and energy metabolism and are key investigational targets for the treatment of several metabolic disorders including insulin resistance, obesity, and type 2 diabetes (T2D) [18]. GLP-1 mimetics have been used clinically for over 10 yr [19], but with the current sharp rise in the worldwide incidence of both T2D and obesity [20], there is a pressing need to develop more effective drugs with fewer associated side effects. We [21,22] and others [23] have previously described how GLP-1R signaling responses are modulated by intracellular membrane trafficking processes, with “biased” agonism [24] being one potentially tractable means to achieve this in a therapeutic context. In the present study, we focus on the influence of the lipid microenvironment on GLP-1R trafficking and functionality in pancreatic beta cells, where it serves to augment glucose-stimulated insulin release to allow tight regulation of blood glucose [25]. We establish here that binding of GLP-1R to the therapeutic agonist exendin-4 (exenatide) triggers increased GLP-1R clustering and reduced lateral diffusion at the plasma membrane, resulting in the segregation of biologically active GLP-1Rs into cholesterol-rich plasma membrane nanodomains that enable compartmentalization of acute receptor signaling responses prior to GLP-1R endocytosis via clathrin-coated pits (CCPs). Interestingly, these processes could be modulated using biased agonists previously derived by us from exendin-4 [22], as well as with the small molecule allosteric modulator 4-(3-benzyloxyphenyl)-2-ethylsulfinyl-6-(trifluoromethyl)pyrimidine (BETP) [26,27]. Moreover, we found that raft localization of the structurally and functionally related glucose-dependent insulinotropic polypeptide receptor (GIPR), also found in beta cells, was constitutive and not subject to agonist-mediated regulation. Disruption of the plasma membrane microarchitecture via cholesterol depletion had a substantial impact on acute GLP-1R clustering, signaling, and endocytosis. We also demonstrate that palmitoylation of the GLP-1R, known to occur at cysteine 438 near the C-terminal end of its cytoplasmic tail [28], is an agonist-mediated event that also plays a role in determining the degree of receptor clustering, nanodomain segregation, and internalization.

This is, to our knowledge, the first report linking receptor palmitoylation and nanodomain partitioning to modified or biased responses of a class B GPCR in pancreatic beta cells, opening the door for future studies based on the direct manipulation of these processes to control GLP-1R action, with the potential to translate into more effective diabetes therapies.

## Results

### Activated GLP-1Rs accumulate in plasma membrane nanodomains

**Fig 1:**
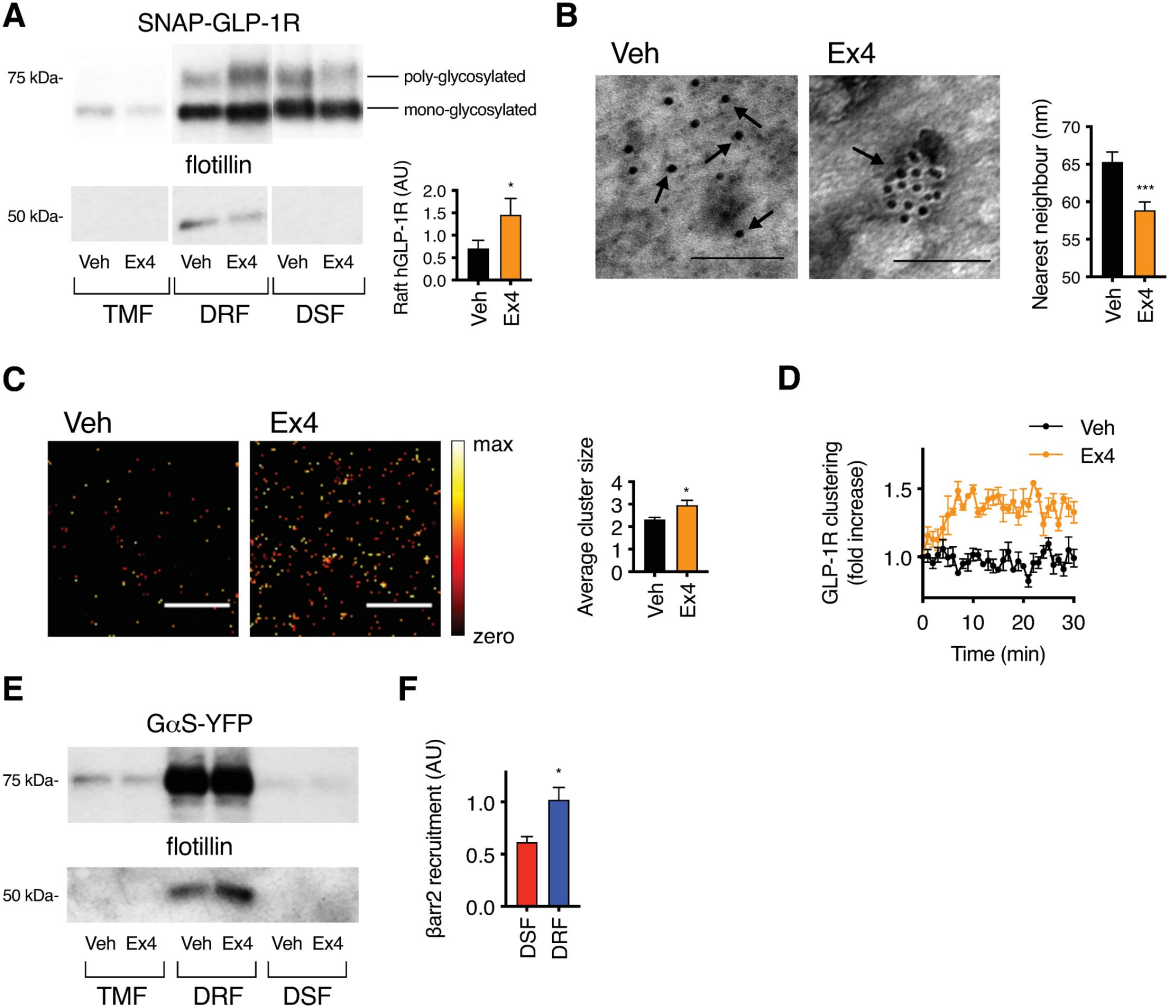
Agonist-induced SNAP-GLP-1R clustering and recruitment to membrane nanodomains. (A) SNAP-GLP-1R distribution within TMFs, DRFs, and DSFs isolated from MIN6B1 cells stably expressing SNAP-GLP-1R treated with vehicle (“Veh”) or 100 nM exendin-4 (“Ex4”) for 2 min, with flotillin as a marker of membrane raft enrichment. Inset shows quantification of poly-glycosylated SNAP-GLP-1R levels in DRFs; individual results normalized to flotillin (raft loading control) and expressed in AU, *n* = 6, paired *t* test. (B) Electron micrographs of gold-labeled SNAP-GLP-1Rs (arrows) from 2D plasma membrane sheets isolated from MIN6B1 cells stably expressing SNAP-GLP-1R following SNAP-tag gold labeling and treatment with vehicle or 100 nM exendin-4 for 2 min. An example of a receptor cluster is shown for the exendin-4-treated condition. Size bars, 100 nm. Mean distances to the nearest neighbor, quantified from a minimum of *n* = 1,000 gold particles per condition, are shown, unpaired *t* test. (C) Representative heatmaps of plasma membrane FLAG-GLP-1R localization following TIRF-PALM imaging and reconstruction with QuickPALM plugin (Fiji) in HEK293 cells stably expressing FLAG-GLP-1Rs labeled with anti-FLAG CAGE 500 antibody prior to stimulation with vehicle or 100 nM exendin-4 for 2 min. Size bars, 1 μm. Average cluster sizes from *n* = 11 regions per condition, paired *t* test. (D) Agonist-induced SNAP-GLP-1R clustering in INS-1 832/3 GLP-1R^−/−^ SNAP-GLP-1R cells dual-labeled with Lumi4-Tb and SNAP-Surface 647, treated with vehicle or 100 nM exendin-4, TR-FRET displayed as fold increase relative to individual baseline, *n* = 4. (E) GαS-YFP distribution within TMFs, DRFs, and DSFs isolated from MIN6B1 SNAP-GLP-1R cells transiently transfected with GαS-YFP prior to treatment with vehicle or 100 nM exendin-4 for 2 min, with flotillin as a marker of membrane raft enrichment. (F) Level of β-arrestin-2 (“βarr2”) recruitment to the GLP-1R in DSFs versus DRFs of CHO-PathHunter (DiscoverX) cells expressing ProLink-tagged human GLP-1R and β-arrestin-2-EA. β-arrestin-2 recruitment was calculated as fold increase of exendin-4 over vehicle condition, normalized to GLP-1R levels in each membrane fraction, and expressed as the fraction of total β-arrestin-2 recruitment for each experimental repeat, *n* = 4, paired *t* test. *p < 0.05, ***p < 0.001, by statistical test indicated in the text. All data are shown as mean ± SEM. Underlying raw data for all the panels included in this figure can be found in S1 Data, and uncropped blots from this figure can be found in S1 Raw Images. AU, arbitrary unit; DRF, detergent-resistant fraction; DSF, detergent-soluble fraction; GαS, Gs alpha subunit; GLP-1R, glucagon-like peptide-1 receptor; HEK293, human embryonic kidney 293; PALM, photoactivatable localization microscopy; TIRF, total internal reflection fluorescence; TMF, total membrane fraction; TR-FRET, time-resolved Förster resonance energy transfer; YFP, yellow fluorescent protein.

Association with plasma membrane raft nanodomains is a known mechanism to organize membrane receptors into signaling microclusters [29]. Localization of GPCRs to membrane rafts can be constitutive or ligand-dependent [30]. To determine the influence of agonist binding upon GLP-1R raft partitioning in pancreatic beta cells, we measured SNAP-GLP-1R levels in purified detergent-resistant fractions (DRFs) and detergent-soluble fractions (DSFs) from total membrane preparations of mouse insulinoma MIN6B1 cells [31] stably expressing human GLP-1R SNAP-tagged at the extracellular N terminus (MIN6B1 SNAP-GLP-1R cells) [21]. Stimulation with a saturating concentration of the orthosteric peptide GLP-1R agonist exendin-4 resulted in increased receptor recruitment to flotillin-positive DRFs compared to vehicle control conditions, suggesting increased partitioning into membrane nanodomains (Fig 1A). The increased raft partitioning primarily concerned the poly-glycosylated fraction of the receptor [32] (S1A Fig), known to represent the receptor pool functional for agonist binding at the cell surface [33,34] (S1B Fig). Increased enrichment of poly-glycosylated GLP-1Rs in membrane raft fractions was also present at lower concentrations of exendin-4 and upon stimulation with the endogenous agonist GLP-1(7–36)NH_2_ (S1C Fig). To corroborate this biochemical finding, we developed a new method to monitor SNAP-GLP-1R localization in cholesterol-rich nanodomains using time-resolved (TR)-Förster resonance energy transfer (FRET) and the solvatochromic membrane probe NR12S [35], as the latter shows blue-shifted emission in liquid-ordered compared to liquid-disordered membrane environments (S1D Fig). In cells expressing SNAP-GLP-1R labeled with the long-lived lanthanide FRET donor Lumi4-Tb, TR-FRET was increased upon exendin-4 addition (S1E Fig), indicating relative movement between the Lumi4-Tb-labeled GLP-1R extracellular domain and the plasma membrane, possibly reflecting the closure of the receptor extracellular domain upon ligand binding, as recently suggested [36]. This signal increase was preferentially detected at the blue-shifted, liquid-ordered–associated part of the spectrum (S1F–S1H Fig), consistent with our earlier observation that exendin-4 stimulation causes SNAP-GLP-1R translocation into detergent-resistant membrane nanodomains.

As raft association is known to regulate cell surface receptor clustering [8], we next determined the degree of GLP-1R clustering at the plasma membrane before and after stimulation with exendin-4. We performed electron microscopy (EM) analysis of intact 2D sheets of apical membranes ripped off from adherent MIN6B1 SNAP-GLP-1R cells labeled live with the membrane-impermeable BG-SS-PEG4-biotin SNAP-tag probe followed by gold-conjugated streptavidin (Fig 1B, S1I Fig). Quantification of gold-particle distances to the nearest neighbor showed increased clustering of SNAP-GLP-1Rs following exendin-4 stimulation. This was supported by total internal reflection fluorescence (TIRF)-photoactivatable localization microscopy (PALM) data, which showed an increase in the average number of receptors per cluster after 1 min of exendin-4 stimulation (Fig 1C), with receptor oligomers already present in basal conditions in keeping with a previous report [37]. To gain more kinetic information on agonist-induced GLP-1R clustering, we used a dual surface labeling approach that allows detection of receptor–receptor interactions by TR-FRET. Because of the long-lived fluorescence of Lumi4-Tb, energy transfer in this assay results from both stable and transient protein–protein interactions, which increases when receptors are in closer proximity [38,39]. Using rat insulinoma INS-1 832/3 cells engineered by clustered regularly interspaced short palindromic repeat (CRISPR)/CRISPR-associated 9 (Cas9) to delete endogenous GLP-1R [40] stably expressing human SNAP-GLP-1R (INS-1 832/3 GLP-1R^−/−^ SNAP-GLP-1R cells), we detected a rapid increase in TR-FRET with exendin-4, which reached a maximum within 10 min (Fig 1D).

Membrane nanodomains have often been described as hotspots for signaling as a result of cosegregation of receptors with their corresponding signaling effectors [5]. Using MIN6B1 SNAP-GLP-1R cells expressing the yellow fluorescent protein (YFP)-tagged Gs alpha subunit (Gα_S_), we found that Gα_S_-YFP clearly partitioned into DRFs both before and after exendin-4 stimulation (Fig 1E). Moreover, exendin-4-induced recruitment of the active GPCR regulatory factor β-arrestin-2 [41] to the GLP-1R was significantly increased in DRFs versus DSFs of Chinese hamster ovary (CHO)-PathHunter cells expressing ProLink (PK)-tagged human GLP-1R and β-arrestin-2-EA (Fig 1F).

### Effect of methyl-β-cyclodextrin-induced nanodomain disruption on GLP-1R behavior

**Fig 2:**
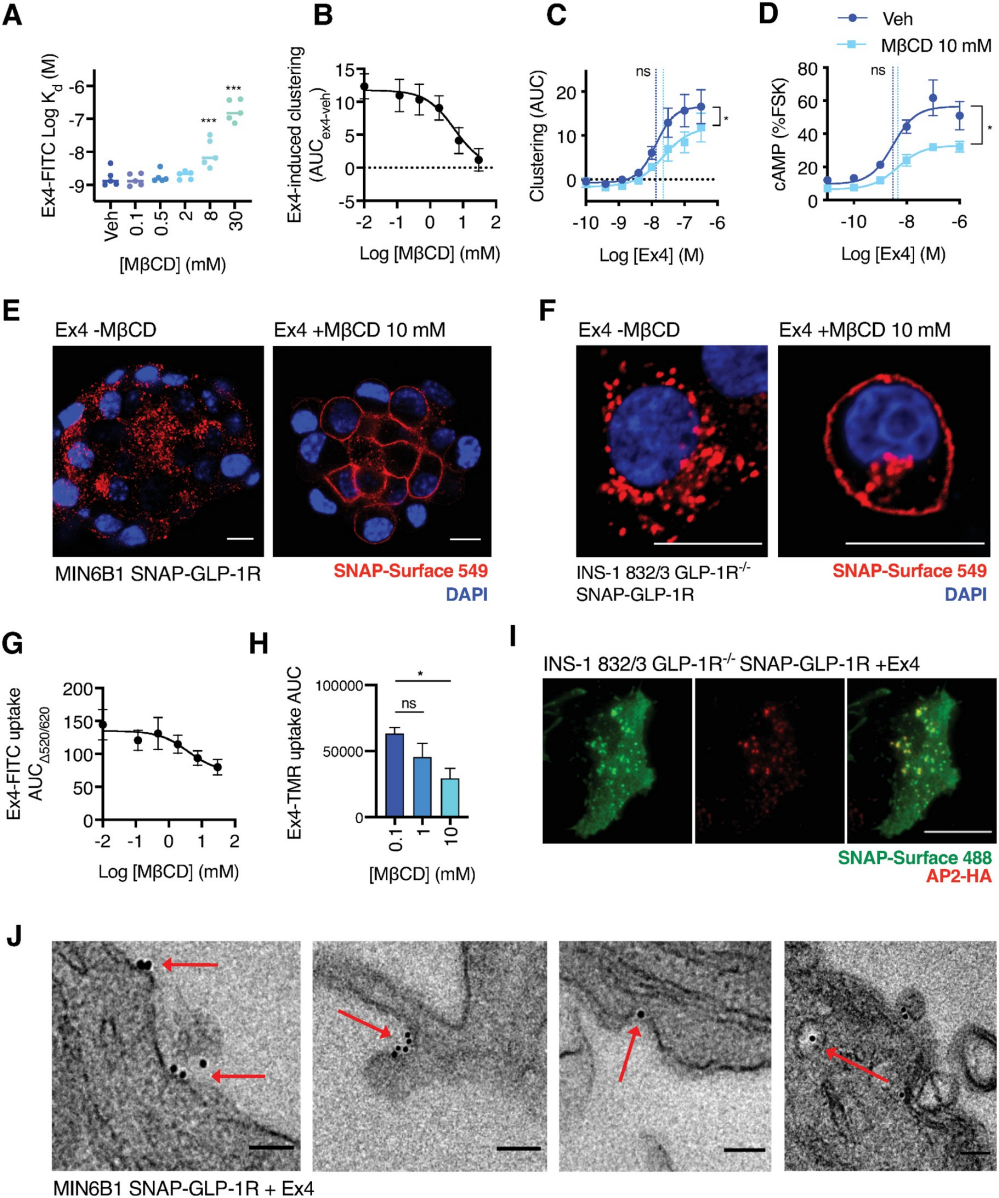
Inhibition of nanodomain compartmentalization reduces GLP-1R signaling and trafficking responses. (A) Equilibrium binding affinity measurements for exendin-4 (“Ex4”)-K12-FITC in INS-1 832/3 GLP-1R^−/−^ SNAP-GLP-1R cells pretreated with indicated concentration of MβCD, *n* = 5, one-way randomized block ANOVA with Dunnett’s test versus vehicle. (B) Effect of 45-min cholesterol depletion with indicated MβCD concentration on SNAP-GLP-1R clustering induced by 100 nM exendin-4 in INS-1 832/3 GLP-1R^−/−^ SNAP-GLP-1R cells, expressed as the difference between AUC for exendin-4 versus vehicle-induced signal over 30 min, *n* = 5, 3-parameter logistic fit of pooled data shown. (C) Dose-response curves for exendin-4-induced clustering in INS-1 832/3 GLP-1R^−/−^ SNAP-GLP-1R cells with or without cholesterol depletion with MβCD (10 mM, 45 min), measured by HTRF and quantified as AUC for each concentration tested, 4-parameter logistic fit of pooled data shown and used to quantify E_max_ and log EC_50_ (vertical dotted lines), paired *t* tests performed on parameter estimates from *n* = 5 experimental repeats. HTRF traces shown in S2H Fig. (D) cAMP dose response to exendin-4 in wt INS-1 832/3 cells, 10-min stimulation with 500 μM IBMX, normalized to FSK response (10 μM), 4-parameter logistic fit of pooled data shown and used to quantify E_max_ and log EC_50_ (vertical dotted lines), paired *t* tests performed on parameter estimates from *n* = 5 experimental repeats. (E) Confocal analysis of SNAP-GLP-1R internalization in MIN6B1 SNAP-GLP-1R cells labeled with SNAP-Surface 549 probe (red) for 30 min and then incubated with or without MβCD (10 mM, 1 h) before stimulation with 100 nM exendin-4 for 15 min. Nuclei (DAPI), blue; size bars, 10 μm. (F) As in (E) but with INS-1 832/3 GLP-1R^−/−^ SNAP-GLP-1R cells. (G) Effect of indicated concentration of MβCD on uptake of exendin-4-K12-FITC in INS-1 832/3 GLP-1R^−/−^ SNAP-GLP-1R cells, expressed as AUC relative to baseline for each concentration, *n* = 5, 3-parameter logistic fit of pooled data shown. (H) Level of exendin-4-K12-TMR uptake in INS-1 832/3 GLP-1R^−/−^ SNAP-GLP-1R cells, calculated as AUC of time-lapse confocal microscopy kinetic traces (shown in S2M Fig) after pretreatment with the indicated MβCD concentration for 1 h, one-way ANOVA with Dunnett’s test, *n* = 6 traces analyzed from three time-lapse acquisitions per condition. (I) TIRF plasma membrane microscopy analysis of INS-1 832/3 GLP-1R^−/−^ SNAP-GLP-1R cells transiently transfected with μ2-HA-WT, which codes for the μ2 domain of the clathrin adaptor AP2 fused to an HA tag (AP2-HA, red), labeled with SNAP-Surface 488 (green) and stimulated for 1 min with 100 nM exendin-4 prior to fixation and HA-tag immunofluorescence; size bar, 10 μm. (J) Representative electron micrographs depicting different stages of CCP endocytosis of gold-labeled SNAP-GLP-1Rs (red arrows) in MIN6B1 SNAP-GLP-1R cells stimulated for 1 min with 100 nM exendin-4; size bars, 100 nm. *p < 0.05, ***p < 0.001, “ns” indicates nonsignificant, by statistical test indicated in the text. All data are shown as mean ± SEM, with individual replicates indicated where relevant. Underlying raw data for all the panels included in this figure can be found in S1 Data, and a dose-response summary for this figure is included in S1 Table. AP2, adaptor protein 2; AUC, area under the curve; cAMP, cyclic adenosine monophosphate; CCP, clathrin-coated pit; FITC, fluorescein isothiocyanate; FSK, forskolin; GLP-1R, glucagon-like peptide-1 receptor; HA, hemagglutinin; HTRF, homogenous time-resolved fluorescence; IBMX, isobutylmethylxanthine; MβCD, methyl-β-cyclodextrin; TIRF, total internal reflection fluorescence; wt, wild type.

To investigate the effect of nanodomain recruitment on GLP-1R cellular behavior in beta cells, we used methyl-β-cyclodextrin (MβCD) to disrupt the plasma membrane architecture via sequestration of cholesterol and other lipids [42]. Efficiency of cholesterol depletion by MβCD was determined by filipin staining and quantified biochemically; note also that MβCD treatment did not alter receptor surface SNAP-GLP-1R expression (S2A–S2C Fig). We observed that binding affinity of the fluorescent exendin-4 conjugate exendin-4-K12-fluorescein isothiocyanate (FITC) [22] to SNAP-GLP-1R at equilibrium was reduced by MβCD in a dose-dependent manner, as measured by TR-FRET [43] (Fig 2A, S2D Fig). Kinetic binding studies suggested that this was due to faster agonist dissociation (S2E and S2F Fig). Displaying a similar dependency on the concentration of MβCD used, nanodomain disruption also reduced maximal exendin-4-induced GLP-1R clustering (Fig 2B and 2C, S2G Fig). MβCD treatment was also effective in reducing both basal and exendin-4-induced NR12S-linked increases in TR-FRET from the liquid-ordered-associated portion of the spectrum (S2H Fig), as expected from its lipid raft-disrupting properties.

Membrane nanodomains have also been implicated in coordination of receptor signaling and endocytosis [44]. In keeping with this, coupling of endogenously expressed GLP-1R in wild-type INS-1 832/3 cells to cyclic adenosine monophosphate (cAMP) production was reduced by MβCD (Fig 2D), with both FRET-based conformational biosensor measurements of cAMP production using ^T^Epac^VV^ [45] and raft-associated activation of protein kinase A (PKA) using AKAR4-Lyn [46], showing a similar pattern (S2I Fig). Additionally, confocal microscopy analysis in beta and nonbeta cells showed a marked inhibition of exendin-4-induced SNAP-GLP-1R internalization after MβCD treatment (Fig 2E and 2F, S2J and S2K Fig, S1 and S2 Movies). Using different techniques to monitor uptake of the fluorescent exendin-4 ligands exendin-4-K12-FITC and exendin-4-K12-5-carboxytetramethylrhodamine (TMR) at different MβCD concentrations in INS-1 832/3 GLP-1R^−/−^ SNAP-GLP-1R cells again showed a similar MβCD dose relationship to that observed for binding affinity and clustering, albeit with some remaining receptor-mediated agonist uptake detected even at maximal doses of MβCD (Fig 2G and 2H, S2L and S2M Fig).

Endocytosis of receptors previously recruited to membrane nanodomains can occur via a range of clathrin-dependent and clathrin-independent pathways, with the specific pathway utilized in each case being particular to each individual receptor [47–50]. We therefore investigated the route of GLP-1R endocytosis by performing TIRF microscopy analysis of INS-1 832/3 GLP-1R^−/−^ SNAP-GLP-1R cells expressing a hemagglutinin (HA)-tagged version of the clathrin adaptor protein 2 (AP2) following 1-min stimulation with exendin-4. We found clustered SNAP-GLP-1Rs greatly colocalized with AP2-HA loci at the plasma membrane (Manders’ coefficient 0.967 ± 0.001) (Fig 2I), indicating that the main pathway of entry for GLP-1Rs in these cells is clathrin-mediated. To support these results, we performed EM analysis of ultrathin sections of MIN6B1 SNAP-GLP-1R cells gold-labeled as in Fig 1. Stimulation with exendin-4 for 1 min resulted in the identification of SNAP-GLP-1R-bound gold particles at different stages of CCP formation (Fig 2J).

**Fig 3:**
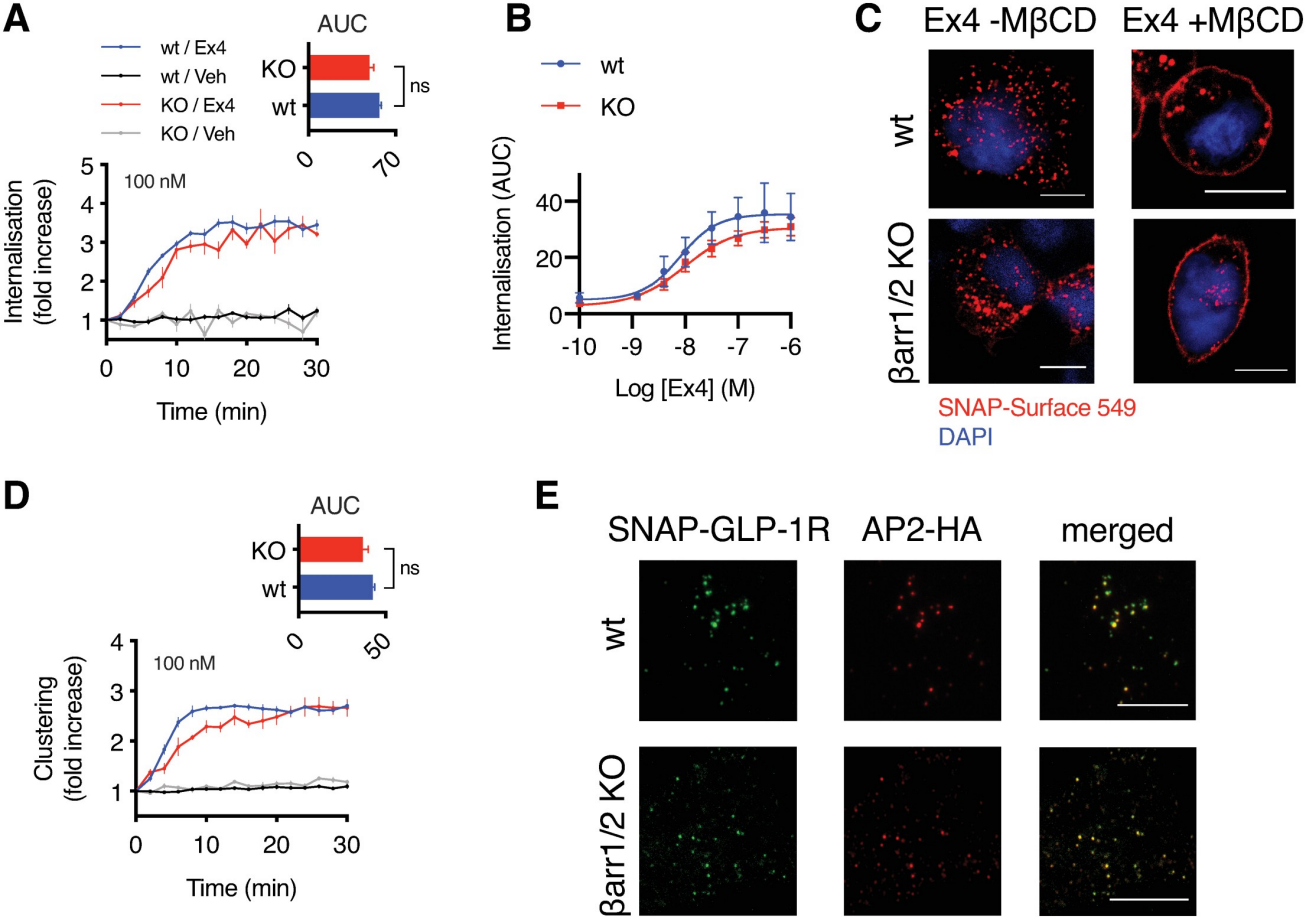
β-arrestins are dispensable for GLP-1R clustering and endocytosis. (A) SNAP-GLP-1R internalization measured by DERET in wt and β-arrestin-less (“KO”) HEK293 cells stably expressing SNAP-GLP-1R, treated with 100 nM exendin-4 or vehicle (“Veh”), expressed relative to baseline, *n* = 4. Inset shows AUC, paired *t* test. (B) Dose responses for SNAP-GLP-1R internalization measured by DERET, analogous experiments to (A), quantified as AUC for each dose tested, 4-parameter logistic fit of pooled data shown, *n* = 4. (C) Confocal analysis of SNAP-GLP-1R internalization in wt and β-arrestin-less HEK293 cells stably expressing SNAP-GLP-1R, labeled with SNAP-Surface 549 (red) and treated with or without MβCD before stimulation with 100 nM exendin-4 for 15 min. Nuclei (DAPI), blue; size bars, 10 μm. (D) SNAP-GLP-1R clustering measured by HTRF in wt and β-arrestin-less HEK293 cells stably expressing SNAP-GLP-1R, treated with 100 nM exendin-4 or vehicle, expressed relative to baseline, *n* = 4. Inset shows AUC, paired *t* test. (E) TIRF microscopy analysis of plasma membranes from wt and β-arrestin-less HEK293 cells stably expressing SNAP-GLP-1R and transiently transfected with AP2-HA, labeled with SNAP-Surface 488 (green) and stimulated for 1 min with 100 nM exendin-4 prior to fixation and HA-tag immunofluorescence (red); size bars, 10 μm, “ns” indicates nonsignificant, by statistical test indicated in the text. All data are shown as mean ± SEM. Underlying raw data for all the panels included in this figure can be found in S1 Data, and a dose-response summary for this figure is included in S2 Table. AP2, adaptor protein 2; AUC, area under the curve; DERET, diffusion-enhanced resonance energy transfer; GLP-1R, glucagon-like peptide-1 receptor; HA, hemagglutinin; HEK293, human embryonic kidney 293; HTRF, homogenous time-resolved fluorescence; MβCD, methyl-β-cyclodextrin; TIRF, total internal reflection fluorescence; wt, wild type.

Recruitment of β-arrestins to active GPCRs has traditionally been considered as a means of receptor desensitization coupled to clathrin-dependent endocytosis [41]. In a previous study, we detected a very transient delay on GLP-1R internalization in cells lacking both β-arrestin-1 and β-arrestin-2, out of keeping with the more marked effects on signaling responses, which we now attribute primarily to avoidance of β-arrestin-mediated desensitization at the plasma membrane [22]. In view of our latest observation of the profound effect of cholesterol depletion on GLP-1R internalization, we reassessed the contribution of β-arrestins with and without MβCD exposure. Using diffusion-enhanced resonance energy transfer (DERET) [51], we first noted very minor differences in agonist-induced SNAP-GLP-1R endocytosis in wild-type versus β-arrestin-1/2 knockout human embryonic kidney 293 (HEK293) cells [52] (Fig 3A and 3B). However, endocytosis was substantially inhibited by MβCD irrespective of β-arrestin status (Fig 3C), suggesting that preservation of membrane nanodomain organization, rather than recruitment of β-arrestins, is critical to sustain SNAP-GLP-1R cell entry. Indeed, exendin-4-induced SNAP-GLP-1R clustering was robust in both cell types (Fig 3D), and we also found similar binding affinities for exendin-4-K12-FITC (S3A and S3B Fig). In view of the persistence of GLP-1R endocytosis in the absence of β-arrestins, and as these are classically thought to couple GPCRs to clathrin via AP2 [53], we again probed the route of GLP-1R endocytosis by TIRF microscopy in wild-type versus β-arrestin-1/2 knockout cells. We found a high degree of colocalization of clustered SNAP-GLP-1Rs with AP2 loci in both cell types (Manders’ coefficients 0.969 ± 0.005 and 0.967 ± 0.002, wild-type and β-arrestin-1/2 knockout, respectively) (Fig 3E). Overall, our results suggest that the main pathway for entry of GLP-1Rs is clathrin-mediated but does not require β-arrestins.

### Role of agonist-mediated palmitoylation in controlling GLP-1R behavior

**Fig 4:**
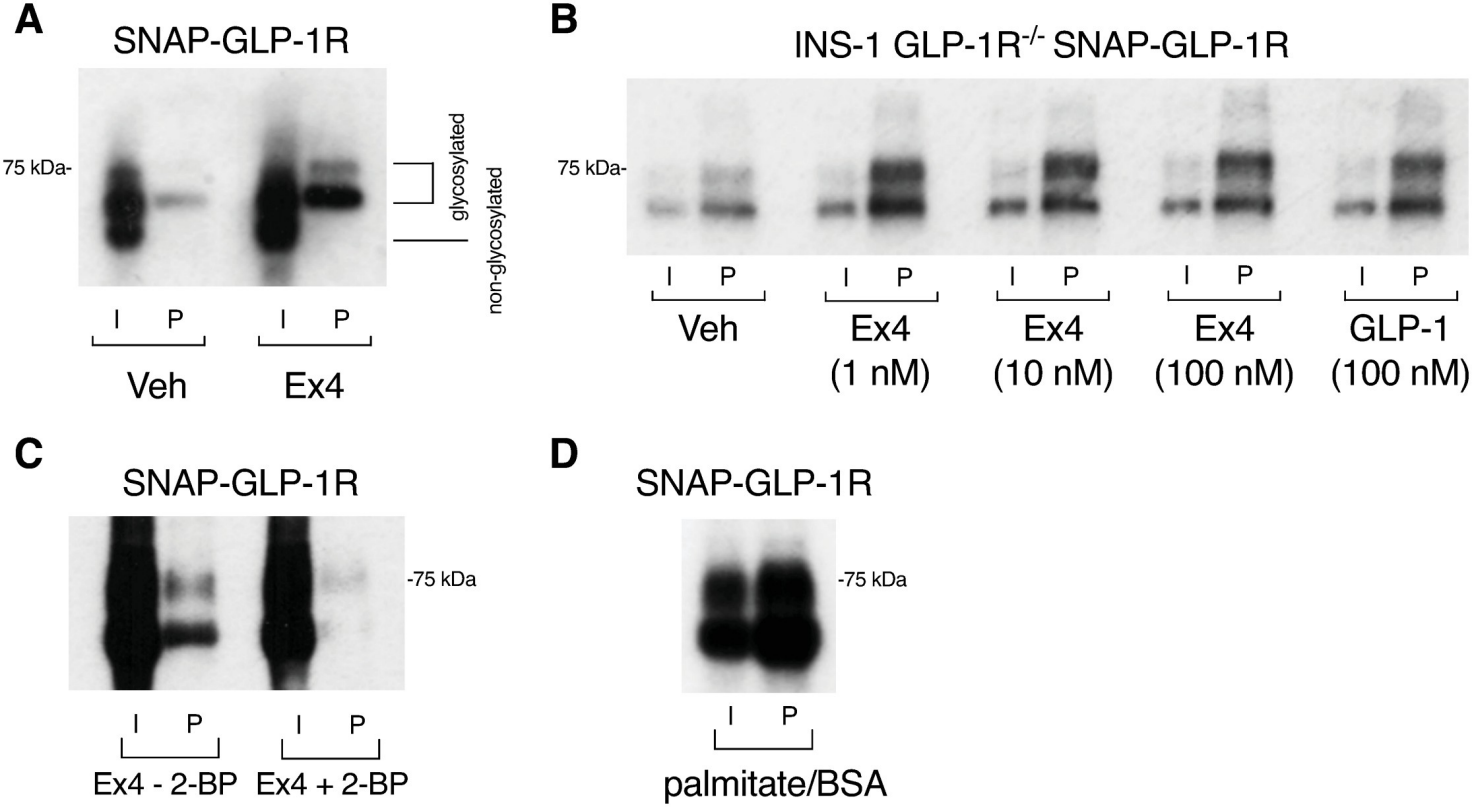
GLP-1R undergoes agonist-induced palmitoylation. (A) Total input (“I”) and palmitoylated (“P”) SNAP-GLP-1R fractions from CHO SNAP-GLP-1R cells treated with vehicle (“Veh”) or 100 nM exendin-4 (“Ex4”) for 10 min. (B) Total input and palmitoylated SNAP-GLP-1R fractions from INS-1 832/3 GLP-1R^−/−^ SNAP-GLP-1R cells treated with vehicle or the indicated concentrations of exendin-4 or GLP-1 for 10 min. (C) Total input and palmitoylated SNAP-GLP-1R fractions from MIN6B1 SNAP-GLP-1R cells treated with 100 nM exendin-4 for 10 min, with and without pretreatment with 200 μM 2-BP overnight. (D) Total input and palmitoylated SNAP-GLP-1R fractions from MIN6B1 SNAP-GLP-1R cells treated with 200 μM palmitate/BSA overnight. Uncropped blots from this figure can be found in S1 Raw Images. 2-BP, 2-bromopalmitate; BSA, bovine serum albumin; CHO, Chinese hamster ovary; GLP-1R, glucagon-like peptide-1 receptor.

C-terminal palmitoylation is one of the means by which GPCRs may partition into membrane rafts [2,16,17]. Prompted by this, and by a previous report demonstrating constitutive palmitoylation of the GLP-1R cytoplasmic tail at cysteine 438 [28], we analyzed the importance of this posttranslational modification in GLP-1R agonist-dependent nanodomain segregation. Using CHO SNAP-GLP-1R cells, we detected a low level of basal GLP-1R palmitoylation that was noticeably increased following exendin-4 stimulation (Fig 4A). To confirm these results in pancreatic beta cells, we repeated the palmitoylation assay in INS-1 832/3 GLP-1R^−/−^ SNAP-GLP-1R cells, where we detected increased GLP-1R palmitoylation following stimulation with a range of exendin-4 concentrations, as well as with the endogenous agonist GLP-1(7–36)NH_2_ (Fig 4B). GLP-1R palmitoylation was also examined in MIN6B1 SNAP-GLP-1R cells following exendin-4 stimulation in the presence or absence of the palmitoylation inhibitor 2-bromopalmitate (2-BP) (Fig 4C) and following incubation with excess palmitate (Fig 4D), where it was greatly enhanced.

**Fig 5:**
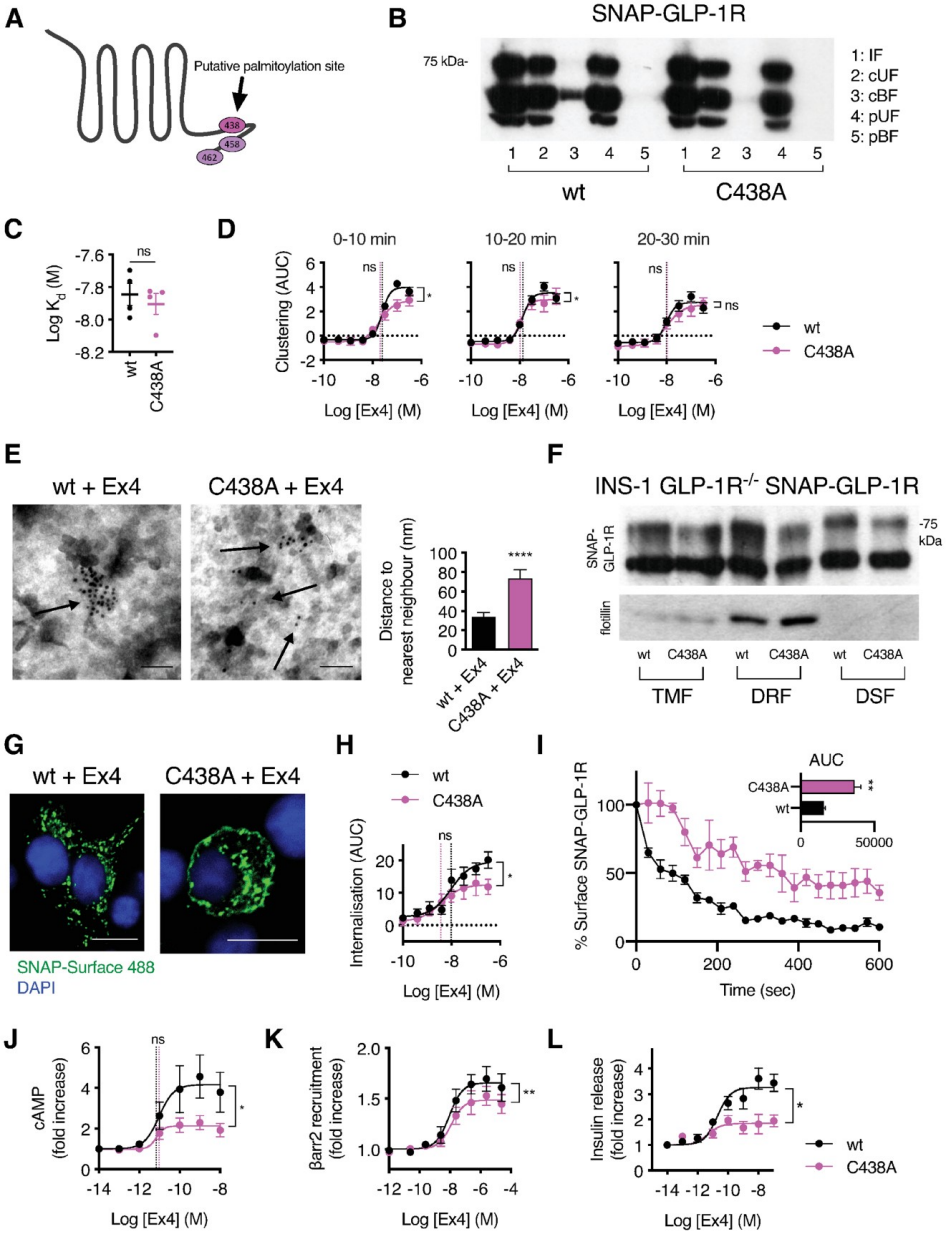
Reduced nanodomain segregation, signaling, and internalization of the palmitoylation-deficient GLP-1R C438A mutant. (A) Cartoon showing C-terminal cysteine residues in the GLP-1R cytoplasmic tail, with C438 highlighted as the putative palmitoylation site. (B) Analysis of SNAP-GLP-1R palmitoylation levels in CHO SNAP-GLP-1R wt or C438A cells treated with 100 nM exendin-4 for 10 min. (C) Equilibrium dissociation binding constant of exendin-4-K12-FITC determined from HTRF kinetic binding experiments in INS-1 832/3 GLP-1R^−/−^ stably expressing wt or C438A SNAP-GLP-1R, *n* = 4, paired *t* test. Binding traces shown in S4C Fig. (D) Dose responses for exendin-4-induced clustering, measured by HTRF in INS-1 832/3 GLP-1R^−/−^ stably expressing wt or C438A SNAP-GLP-1R, expressed as AUC for each concentration tested after segmentation into indicated time windows, 4-parameter logistic fits of pooled data shown with E_max_ and log EC_50_ (vertical dotted lines), paired *t* tests comparing parameter estimates for *n* = 5 repeats. Traces shown in S4E Fig. (E) Representative electron micrographs of gold-labeled SNAP-GLP-1Rs (arrows) from 2D plasma membrane sheets isolated from HEK293 cells stably expressing wt or C438A mutant SNAP-GLP-1R following SNAP-tag gold labeling and treatment with 100 nM exendin-4 for 2 min; size bars, 100 nm. Average distance to the nearest neighbor is shown from a minimum of *n* = 300 gold particles per condition, unpaired *t* test. (F) SNAP-GLP-1R wt versus C438A distribution within TMFs, DRFs, and DSFs isolated from INS-1 832/3 GLP-1R^−/−^ cells stably expressing each type of SNAP-GLP-1R and treated with 100 nM exendin-4 for 2 min, with flotillin as a marker of membrane raft enrichment. (G) Confocal analysis of SNAP-GLP-1R wt versus C438A internalization in INS-1 832/3 GLP-1R^−/−^ cells stably expressing each type of SNAP-GLP-1R following labeling with SNAP-Surface 488 for 30 min and stimulation with 100 nM exendin-4 for 15 min. Nuclei (DAPI), blue; size bars, 10 μm. (H) Dose responses for wt or C438A SNAP-GLP-1R internalization induced by exendin-4 in INS-1 832/3 GLP-1R^−/−^ cells stably expressing each SNAP-receptor type, measured by DERET, quantified as AUC for each concentration tested, 4-parameter logistic fits of pooled data shown with E_max_ and log EC_50_ (vertical dotted lines), paired *t* tests performed on parameter estimates from *n* = 5 repeats. (I) Dynamic internalization profile, assessed as decrease in plasma membrane signal, from time-lapse confocal microscopy data of INS-1 832/3 GLP-1R^−/−^ cells stably expressing wt or C438A SNAP-GLP-1R following labeling with SNAP-Surface 549 for 30 min and stimulation with 100 nM exendin-4, *n* = 4, data normalized to baseline for every individual trace. Inset shows AUC calculated from main graph, unpaired *t* test. (J) Exendin-4-induced cAMP in INS-1 832/3 GLP-1R^−/−^ cells stably expressing wt or C438A SNAP-GLP-1R, 10-min exendin-4 stimulation with 500 μM IBMX, *n* = 6, 3-parameter fits shown and used to quantify E_max_ and log EC_50_ (vertical dotted lines), paired *t* tests. (K) Dose-response curves of β-arrestin-2 recruitment to the GLP-1R in HEK293 β-arrestin-2-EA cells transiently transfected with wt or C438A SNAP-GLP-1R-PK, normalized to basal response, *n* = 5, E_max_ compared by paired *t* test. (L) Exendin-4-induced insulin secretion in INS-1 832/3 GLP-1R^−/−^ cells stably expressing wt or C438A SNAP-GLP-1R, 16-h stimulation at 11 mM glucose, expressed relative to 11 mM glucose alone, 3-parameter fits of pooled data shown, E_max_ from *n* = 4 repeats compared by paired *t* test. *p < 0.05, **p < 0.01, ****p < 0.0001, “ns” indicates nonsignificant, by statistical test indicated in the text. All data are shown as mean ± SEM, with individual replicates indicated where relevant. Underlying raw data for all the panels included in this figure can be found in S1 Data, and a dose-response summary for this figure is included in S3 Table; uncropped blots from this figure can be found in S1 Raw Images. AUC, area under the curve; cAMP, cyclic adenosine monophosphate; cBF, cleaved bound fraction (corresponding to the palmitoylated pool); CHO, Chinese hamster ovary; cUF, cleaved unbound fraction; DERET, diffusion-enhanced resonance energy transfer; DRF, detergent-resistant fraction; DSF, detergent-soluble fraction; FITC, fluorescein isothiocyanate; GLP-1R, glucagon-like peptide-1 receptor; HEK293, human embryonic kidney 293; HTRF, homogenous time-resolved fluorescence; IF, input fraction; pBF, preserved bound fraction; pUF, preserved unbound fraction; TMF, total membrane fraction; wt, wild type.

In order to further analyze the specific role of GLP-1R palmitoylation on exendin-4-triggered cellular effects, we mutated SNAP-GLP-1R to replace cysteine 438, previously described as its only palmitoylation site [28], with alanine (C438A; Fig 5A). We first confirmed that mutant SNAP-GLP-1R C438A is no longer palmitoylated following exendin-4 stimulation (Fig 5B). Surface levels of both wild-type and C438A SNAP-GLP-1Rs were similar when expressed stably or transiently (S4A and S4B Fig), suggesting that constitutive palmitoylation is not required for efficient delivery of the GLP-1R to the plasma membrane, unlike for some other GPCRs [54]. Additionally, we did not detect any significant differences in binding affinity of exendin-4-K12-FITC to mutant versus wild-type GLP-1R (Fig 5C, S4C and S4D Fig).

In keeping with a putative role for palmitoylation in the formation of receptor clusters, we observed delayed exendin-4-triggered clustering with C438A SNAP-GLP-1R compared to wild type, albeit with a full response eventually appearing after longer agonist exposure (Fig 5D, kinetic traces shown in S4E Fig). Moreover, EM analysis of nearest neighbors in 2D plasma membrane sheets showed reduced exendin-4-induced clustering with the C438A mutant (Fig 5E). Recruitment of poly-glycosylated C438A SNAP-GLP-1Rs to DRFs after exendin-4 stimulation was concomitantly reduced with C438A compared to wild-type receptor (fold increase versus wild type of 0.57 ± 0.02) (Fig 5F).

We next compared the trafficking properties of wild-type versus C438A mutant SNAP-GLP-1R stably expressed in INS-1 832/3 GLP-1R^−/−^, as well as in MIN6B1 cells in which endogenous GLP-1R was knocked out by CRISPR/Cas9 (S4F Fig). We detected reduced internalization of the C438A mutant in both beta cell lines (Fig 5G, S4G Fig), with no effect on receptor recycling after internalization (S4H and S4I Fig). Internalization measurements by DERET or time-lapse confocal microscopy of wild-type or C438A SNAP-GLP-1Rs in INS-1 832/3 GLP-1R^−/−^ cells again revealed reduced internalization with the palmitoylation-deficient mutant (Fig 5H and 5I, S4J Fig, S3 and S4 Movies).

The GLP-1R C438A mutation has variously been reported to result in reduced cAMP signaling [28,55] or to have no effect [56], when measured in HEK293 or CHO-K1 cells. In accordance with the former reports, we found that cAMP production was reduced with C438A compared to wild-type SNAP-GLP-1R in INS-1 832/3 GLP-1R^−/−^ cells (Fig 5J). Consistent with this finding, exendin-4-induced recruitment of β-arrestin-2 to C438A SNAP-GLP-1Rs was reduced compared to wild type (Fig 5K). Critically, this translated into significant reductions in exendin-4-induced insulin secretion (Fig 5L), indicating that GLP-1R palmitoylation may play an important role in glucose homeostasis or responses to pharmacological GLP-1R agonists in diabetes.

The closely related GIPR is also found in beta cells and plays a similar role to GLP-1R in regulating glucose-stimulated insulin release [25]. Interestingly, GIPR C-terminal cysteine residues 406 and 411 have previously been implicated in GIPR desensitization, although palmitoylation was not examined [57]. Highlighting that agonist-dependent modulation of palmitoylation and recruitment to membrane nanodomains are receptor-specific responses, we found that GIPR displayed a high degree of constitutive palmitoylation, which was not further increased by GIP stimulation (S5A Fig). Similarly, SNAP-GIPR was extensively recruited to DRFs under basal conditions (S5B Fig) and showed a higher degree of constitutive clustering compared to GLP-1Rs, despite similar surface expression levels, with no detectable increase upon GIP addition (S5C Fig). Although not agonist-regulated, GIPR localization to cholesterol-rich nanodomains was functionally important, as its endocytosis was virtually abolished after MβCD treatment (S5D Fig).

### Biased GLP-1R agonists show distinct patterns of clustering and palmitoylation

We have previously investigated the effects of a panel of exendin-4-derived biased GLP-1R agonists with marked differences in binding kinetics, preference for cAMP versus β-arrestin recruitment, and receptor internalization and recycling [22]. Although they were measured acutely, bias profiles were predictive of the ability of each agonist to support long-term GLP-1R signaling and insulin secretion from beta cells, with lower-affinity, slow-internalizing agonists more efficacious for longer-term responses despite being less potent acutely. Given that differential palmitoylation and/or nanodomain partitioning has been linked to the establishment of signal bias [58], we explored the possibility that two exemplar agonists from this panel with opposite bias effects, namely exendin-phe1 and exendin-asp3, might influence GLP-1R palmitoylation, clustering, and/or recruitment to membrane nanodomains compared to the parental agonist exendin-4.

**Fig 6:**
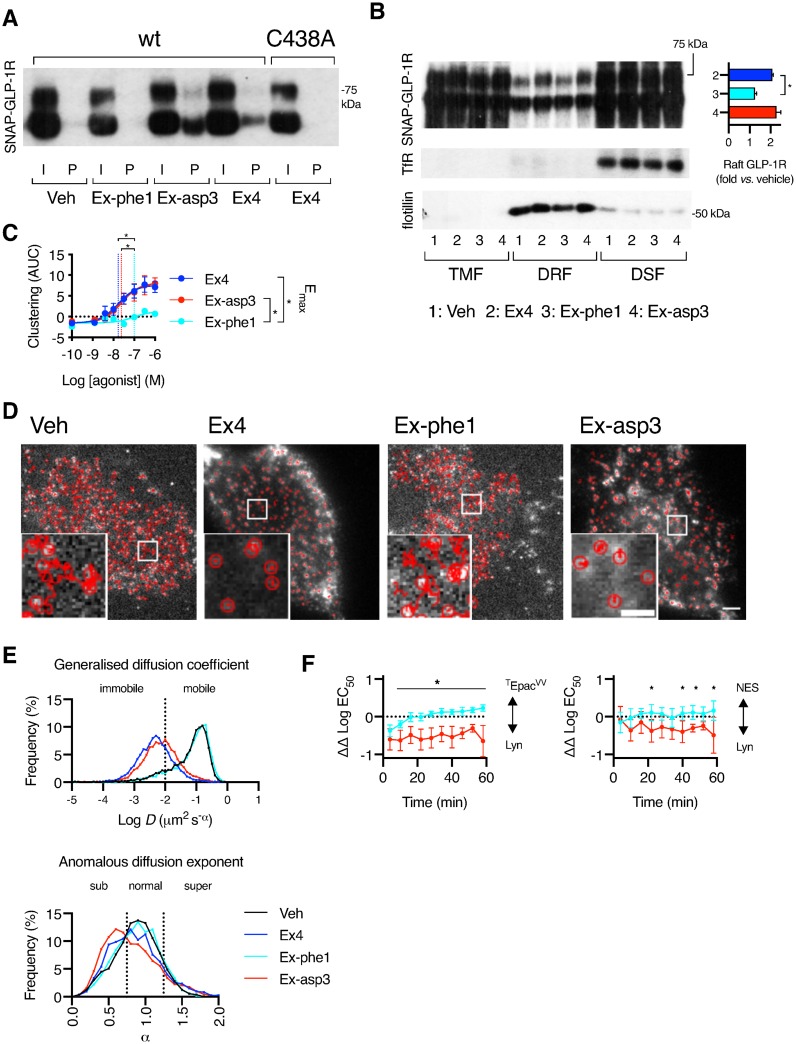
GLP-1R palmitoylation, nanodomain clustering, and spatial control of signaling with biased agonists. (A) Total input (“I”) and palmitoylated (“P”) SNAP-GLP-1R fractions from CHO-K1 cells stably expressing SNAP-GLP-1R wild type or C438A and treated with vehicle (“Veh”) or 100 nM of exendin-4 (“Ex4”), exendin-phe1 (“Ex-phe1”), or exendin-asp3 (“Ex-asp3”) for 10 min. (B) SNAP-GLP-1R distribution within TMFs, DRFs, and DSFs isolated from MIN6B1 SNAP-GLP-1R cells treated with vehicle or indicated agonist (100 nM) for 2 min, with TfR as a marker of membrane non-raft and flotillin as a marker of membrane raft enrichment. Inset shows quantification of poly-glycosylated SNAP-GLP-1R levels in DRFs, with individual results normalized to flotillin (raft loading control), *n* = 3, one-way repeat-measures ANOVA with Dunnett’s test versus exendin-4. (C) Dose responses showing SNAP-GLP-1R clustering induced by each agonist in INS-1 GLP-1R^−/−^ SNAP-GLP-1R cells, expressed as AUC for each concentration tested, 4-parameter logistic fits of pooled data shown with E_max_ and log EC_50_ (vertical dotted lines), one-way repeat-measures ANOVA with Tukey’s test comparing parameter estimates from *n* = 5 repeats. Traces shown in S6E Fig. (D) Representative snapshots of single-molecule TIRF image sequences acquired from plasma membranes of CHO-K1 cells transiently expressing SNAP-GLP-1R labeled with SNAP-Surface 549 prior to stimulation with vehicle or 100 nM of the indicated agonist, including overlaid individual trajectories (red). Size bars, 5 μm, and 2 μm for insets. (E, top) Frequency of immobile versus mobile single-molecule SNAP-GLP-1R trajectories following the indicated treatment, based on the generalized diffusion coefficient D. (E, bottom) Frequency of subdiffusion, normal diffusion, and superdiffusion of single-molecule SNAP-GLP-1Rs following the indicated treatment, based on the anomalous diffusion exponent α. Trajectories (6,100–11,000 for each group) analyzed with u-track (MATLAB) and further categorized using custom algorithms (see Materials and methods section). (F) Summary of relative potency changes in CHO SNAP-GLP-1R cells treated with the indicated agonist measured using the FRET biosensors ^T^Epac^VV^, AKAR4-NES, and AKAR4-Lyn. Bias was determined using the relative potency ratio approach by first subtracting log EC_50_ values for exendin-phe1 and exendin-asp3 from that of exendin-4 and then normalizing values for AKAR4-Lyn from those of AKAR4-NES or ^T^Epac^VV^ generated in the same experiment for each agonist, *n* = 5, two-way repeat-measures ANOVA with Sidak’s test comparing exendin-phe1 with exendin-asp3. For further details, see Materials and methods. Traces are shown in S7A and S7B Fig. *p < 0.05 by statistical test indicated in the text. All data are shown as mean ± SEM. Underlying raw data for all the panels included in this figure can be found in S1 Data, and a dose-response summary for this figure is included in S4 Table; raw trajectory coordinates and MSDs used to calculate generalized diffusion coefficient D and anomalous diffusion exponent α in (E) can be downloaded from https://doi.org/10.6084/m9.figshare.c.4592000; uncropped blots from this figure can be found in S1 Raw Images. AUC, area under the curve; CHO, Chinese hamster ovary; DRF, detergent-resistant fraction; DSF, detergent-soluble fraction; FRET, Förster resonance energy transfer; GLP-1R, glucagon-like peptide-1 receptor; MSD, mean squared displacement; TfR, transferrin receptor; TIRF, total internal reflection fluorescence; TMF, total membrane fraction.

We first analyzed the level of SNAP-GLP-1R palmitoylation elicited by these three agonists and found that stimulation with exendin-asp3, a high-affinity agonist biased toward β-arrestin recruitment and internalization, increased SNAP-GLP-1R palmitoylation compared to exendin-4, whereas stimulation with exendin-phe1, a low-affinity agonist biased in opposite directions, had a reduced effect (Fig 6A). We next examined whether a similar pattern was present for membrane raft partitioning and found that a smaller proportion of poly-glycosylated SNAP-GLP-1Rs segregated to DRFs following stimulation with exendin-phe1, with exendin-asp3 triggering a similar level of raft recruitment to exendin-4 (Fig 6B). Supporting this finding, NR12S FRET measurements indicated a greater increase in signal from liquid-ordered versus liquid-disordered spectral regions with exendin-4 and exendin-asp3 in comparison to exendin-phe1 (S6A and S6B Fig). In keeping with this, both SNAP-GLP-1R clustering and internalization were virtually absent with exendin-phe1 compared to either exendin-4 or exendin-asp3 (Fig 6C, S6C–S6E Fig).

In order to further characterize the behavior of individual GLP-1Rs at the plasma membrane following stimulation with these agonists, we performed single-molecule TIRF microscopy tracking experiments to identify individual receptor trajectories [59,60] in CHO-K1 cells transiently expressing SNAP-GLP-1R at low/physiological levels (Fig 6D, S5–S8 Movies). A mean squared displacement (MSD) analysis was used to analyze individual GLP-1R trajectories [60,61]. These were classified into four groups (S6F Fig) based on both their diffusion coefficient (Fig 6E, top) and their anomalous diffusion exponent (Fig 6E, bottom). Under vehicle conditions, 14% of the receptors were immobile, 32% were confined (subdiffusion), 48% followed normal Brownian diffusion, and 6% had directional motion (superdiffusion). Following exendin-4 stimulation, the fraction of immobile receptors increased to 77%. Exendin-asp3 caused very similar changes to exendin-4 (64% of immobile receptors). Conversely, the effect of exendin-phe1 stimulation was very similar to that of vehicle, with only 12% of the receptors immobile and no significant changes upon the distribution of mobile receptors among the different groups, indicating that stimulation with this biased agonist is associated with a different pattern of lateral diffusion of plasma membrane GLP-1Rs.

Next, we determined signaling responses to each biased agonist at multiple doses in real time in CHO SNAP-GLP-1R cells with ^T^Epac^VV^, AKAR4-NES (cytosolic PKA sensor) or AKAR4-Lyn biosensors (S7A Fig), allowing the construction of sequential dose-response curves across the entire stimulation period to observe changes in potency over time (S7B Fig), so as to assess whether the differential membrane raft translocation of GLP-1R by biased agonists controls spatial organization of cAMP signaling. These results suggested selective reductions in nanodomain-specific AKAR4-Lyn signaling with exendin-phe1 compared to exendin-asp3 (Fig 6F), in keeping with the reduced tendency of this agonist to induce GLP-1R raft localization. Although the signal bias profiles broadly matched the measured localization pattern for each agonist from membrane fractionation experiments, agreement was not perfect. In particular, exendin-asp3 displayed enhanced potency for AKAR4-Lyn signaling, despite no evidence of additional nanodomain translocation beyond that achieved by exendin-4. Nevertheless, these data support the hypothesis that, in contrast with exendin-4, exendin-phe1 fails to immobilize GLP-1Rs in cholesterol-rich signaling hotspots.

Our earlier observation that treatment with MβCD reduces binding affinity of exendin-4-K12-FITC (Fig 2) prompted us to question whether agonist-induced GLP-1R clustering might increase the tendency for dissociating agonist molecules to rebind to nearby receptors, contributing to affinity measures [62]. In view of the profound differences in agonist-induced SNAP-GLP-1R clustering with exendin-phe1 versus exendin-4, we compared the affinity-modulating effects of MβCD on exendin-4-K12-FITC and exendin-phe1-K12-FITC in parallel (S7C and S7D Fig) and found that MβCD reduced binding affinity of both ligands to a similar extent. These data suggest that the effect of MβCD on binding kinetics either precedes or is independent of its effect on receptor clustering, as exendin-phe1-driven clustering is minimal. Of note, a number of GPCRs have been found to form direct and functional allosteric interactions with cholesterol [63,64], the selective depletion of which could underlie the observed reduction in affinity independently of wider effects on plasma membrane microarchitecture.

### Allosterical control of GLP-1R nanodomain segregation

**Fig 7:**
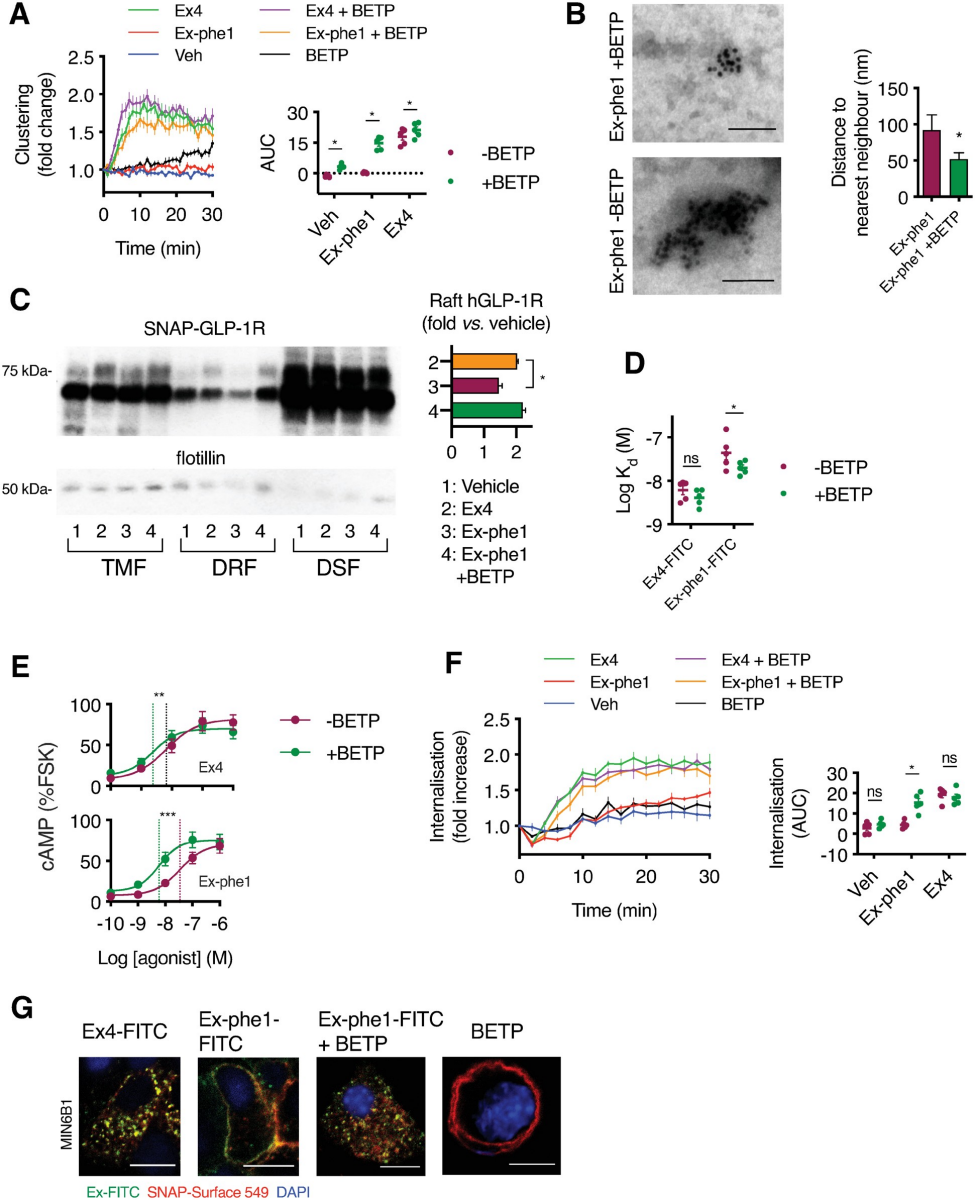
Allosteric control of GLP-1R nanodomain segregation, signaling, and trafficking. (A) SNAP-GLP-1R clustering measured by HTRF in INS-1 GLP-1R^−/−^ SNAP-GLP-1R cells treated with 100 nM exendin-4 (“Ex4”), exendin-phe1 (“Ex-phe1”), or vehicle, ± BETP (10 μM), expressed relative to baseline, *n* = 5. Inset shows AUC for each condition, two-way repeat-measures ANOVA with Sidak’s test comparing each condition ± BETP. (B) Representative electron micrographs of gold-labeled SNAP-GLP-1Rs from 2D plasma membrane sheets isolated from MIN6B1 SNAP-GLP-1R cells following gold labeling of SNAP-tagged receptors and treatment with 100 nM exendin-phe1 with and without BETP (10 μM) for 2 min. Average distance to the nearest neighbor quantified from a minimum of *n* = 155 (− BETP) or *n* = 330 (+ BETP) gold particles per condition, unpaired *t* test. (C) SNAP-GLP-1R distribution within TMFs, DRFs, and DSFs isolated from MIN6B1 SNAP-GLP-1R cells treated with vehicle or 100 nM of the indicated agonist with or without BETP (10 μM) for 2 min, with flotillin as a marker of membrane raft enrichment. Inset shows quantification of poly-glycosylated SNAP-GLP-1R levels in DRFs with individual results normalized to flotillin (raft loading control) shown as vehicle fold increase, *n* = 3, one-way repeat-measures ANOVA with Dunnett’s test versus exendin-4. (D) Effect of BETP (10 μM) on equilibrium dissociation binding constants of exendin-4-K12-FITC and exendin-phe1-K12-FITC in INS-1 GLP-1R^−/−^ SNAP-GLP-1R cells, *n* = 5, two-way repeat-measures ANOVA with Sidak’s test comparing ± BETP. (E) cAMP responses in INS-1 832/3 cells with endogenous GLP-1R expression, stimulated for 10 min in presence of 500 μM IBMX with exendin-4 or exendin-phe1 ± BETP (10 μM), normalized to FSK response (10 μM), 4-parameter logistic fits of pooled data shown with log EC_50_ (vertical dotted lines), two-way repeat-measures ANOVA with Sidak’s test comparing ± BETP from *n* = 5. (F) SNAP-GLP-1R internalization measured by DERET in INS-1 GLP-1R^−/−^ SNAP-GLP-1R cells treated with 100 nM exendin-4, exendin-phe1, or vehicle ± BETP (10 μM), expressed relative to baseline, *n* = 5. Inset shows AUC, two-way repeat-measures ANOVA with Sidak’s test comparing each condition ± BETP. (G) Confocal analysis of the indicated FITC agonist (green) and SNAP-GLP-1R (red) localization in MIN6B1 SNAP-GLP-1R cells following labeling with SNAP-Surface 549 and stimulation with 100 nM of the indicated FITC agonist with or without BETP (10 μM) for 10 min or with BETP alone. Nuclei (DAPI), blue; size bars, 10 μm. *p < 0.05, **p < 0.01, ***p < 0.001, “ns” indicates nonsignificant, by statistical test indicated in the text. All data are shown as mean ± SEM, with individual replicates indicated where relevant. Underlying raw data for all the panels included in this figure can be found in S1 Data, and a dose-response summary for this figure is included in S5 Table; uncropped blots from this figure can be found in S1 Raw Images. AUC, area under the curve; BETP, 4-(3-benzyloxyphenyl)-2-ethylsulfinyl-6-(trifluoromethyl)pyrimidine; cAMP, cyclic adenosine monophosphate; DRF, detergent-resistant fraction; DSF, detergent-soluble fraction; FITC, fluorescein isothiocyanate; FSK, forskolin; GLP-1R, glucagon-like peptide-1 receptor; HTRF, homogenous time-resolved fluorescence; IBMX, isobutylmethylxanthine; TMF, total membrane fraction.

Previous reports highlight that the GLP-1R-specific positive allosteric modulator (PAM) BETP [55] can markedly augment responses to weak GLP-1R agonists such as oxyntomodulin [65] and GLP-1(9–36)NH_2_ [26]. We therefore determined whether BETP played any role in GLP-1R clustering and nanodomain segregation in beta cells. Indeed, whereas the effect of BETP or exendin-phe1 alone on clustering in INS-1 832/3 GLP-1R^−/−^ SNAP-GLP-1R cells was marginal, coapplication of exendin-phe1 with BETP resulted in rapid SNAP-GLP-1R clustering, comparable to that obtained with exendin-4 (Fig 7A). These results were supported by EM analysis from MIN6B1 SNAP-GLP-1R cells stimulated with exendin-phe1 in the presence or absence of BETP (Fig 7B). We also found that BETP was able to rescue the reduced SNAP-GLP-1R segregation to membrane nanodomains elicited by exendin-phe1, with SNAP-GLP-1Rs now being recruited to membrane rafts to the same extent as with exendin-4 (Fig 7C).

Revisiting the concept of receptor cluster-driven rebinding as a possible contributor to agonist binding affinity, we found that costimulation with BETP was able to significantly increase the binding affinity of exendin-phe1-K12-FITC, with a less marked effect on exendin-4-K12-FITC (Fig 7D, S8A and S8B Fig). This agonist specificity correlates with agonist-specific BETP effects on GLP-1R clustering and the putative rebinding mechanism consequent to this, contrasting with the abovementioned agonist-equal affinity changes triggered by MβCD (S7 Fig).

To determine the functional impact of these changes in beta cells, we analyzed the signaling and trafficking characteristics of exendin-phe1 in the presence and absence of BETP. Potency estimates for exendin-phe1-induced cAMP production in INS-1 832/3 cells with endogenous GLP-1R expression were markedly increased, with a smaller effect on potency when exendin-4 was used as the orthosteric probe (Fig 7E). β-arrestin-2 recruitment to the GLP-1R was also substantially increased (S8C Fig). Moreover, DERET measurements showed that SNAP-GLP-1R endocytosis was significantly increased for exendin-phe1 when coapplied with BETP compared to exendin-phe1 alone, with no effect of BETP on vehicle or exendin-4-triggered GLP-1R internalization (Fig 7F). This was supported by confocal microscopy analysis using FITC conjugates of exendin-phe1 ± BETP versus exendin-4 (Fig 7G).

**Fig 8:**
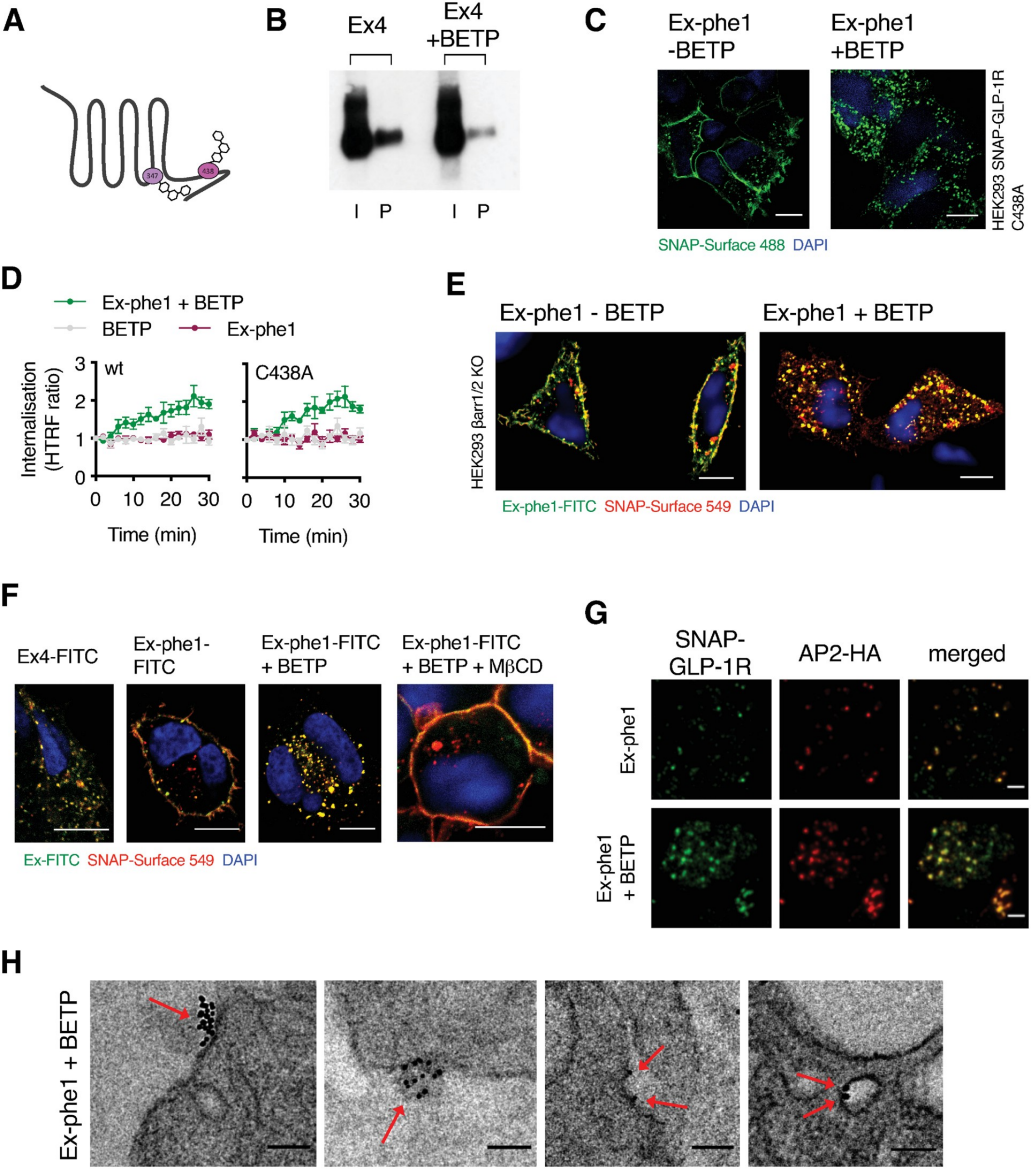
Mechanism of BETP-mediated GLP-1R endocytosis. (A) Cartoon depicting sites of covalent modification of GLP-1R by BETP at C347 and C438, as per [55]. (B) Total input (“I”) and palmitoylated (“P”) SNAP-GLP-1R fractions from CHO SNAP-GLP-1R cells treated with 100 nM exendin-4 (“Ex4”) for 10 min in the presence or absence of 10 μM BETP. (C) Confocal analysis of SNAP-GLP-1R C438A (green) localization in HEK293 cells following labeling with SNAP-Surface 488 and stimulation with exendin-phe1 (“Ex-phe1”) with or without BETP (10 μM) for 10 min. Nuclei (DAPI), blue; size bars, 10 μm. (D) SNAP-GLP-1R internalization measured by DERET in HEK293 cells transiently expressing wt or C438A mutant SNAP-GLP-1R, treated with 100 nM exendin-phe1, 10 μM BETP, or both, expressed as fold increase from baseline, *n* = 6. (E) Confocal analysis of exendin-phe1-K12-FITC (green) and SNAP-GLP-1R (red) internalization in HEK293 β-arrestin-less (“βarr1/2 KO”) cells stably expressing SNAP-GLP-1R following labeling with SNAP-Surface 549 and stimulation with 100 nM of exendin-phe1-K12-FITC for 40 min with or without BETP (10 μM). Nuclei (DAPI), blue; size bars, 10 μm. (F) Confocal analysis of the indicated FITC agonist (green) and SNAP-GLP-1R (red) localization in MIN6B1 SNAP-GLP-1R cells following labeling with SNAP-Surface 549 and stimulation with 100 nM of the indicated FITC agonist with or without BETP (10 μM) for 10 min in the presence or absence of MβCD (10 mM, 1 h preincubation). Nuclei (DAPI), blue; size bars, 10 μm. (G) TIRF microscopy analysis of plasma membranes from MIN6B1 SNAP-GLP-1R cells transiently transfected with AP2-HA, following labeling with SNAP-Surface 488 (green) and stimulation for 1 min with 100 nM exendin-phe1 with and without BETP (10 μM), prior to fixation and HA-tag immunofluorescence (red); size bars, 2 μm. (H) Representative electron micrographs depicting different stages of CCP endocytosis of gold-labeled SNAP-GLP-1Rs (red arrows) in MIN6B1 SNAP-GLP-1R cells stimulated for 1 min with 100 nM exendin-phe1 with BETP (10 μM); size bars, 100 nm. All data are shown as mean ± SEM. Underlying raw data for all the panels included in this figure can be found in S1 Data; uncropped blots from this figure can be found in S1 Raw Images. AP2, activator protein 2; BETP, 4-(3-benzyloxyphenyl)-2-ethylsulfinyl-6-(trifluoromethyl)pyrimidine; CCP, clathrin-coated pit; FITC, fluorescein isothiocyanate; GLP-1R, glucagon-like peptide-1 receptor; HA, hemagglutinin; HEK293, human embryonic kidney 293; MβCD, methyl-β-cyclodextrin; TIRF, total internal reflection fluorescence; wt, wild type.

To further elucidate the mechanism by which BETP potentiates GLP-1R responses, we examined the relative contributions of GLP-1R palmitoylation, β-arrestins, and cholesterol-dependent membrane compartmentalization to its effect. BETP is known to cross the plasma membrane to covalently bind two intracellular GLP-1R cysteine residues (347 and 438, see Fig 8A for a schematic of BETP GLP-1R binding sites) [55]. Accordingly, BETP caused a significant reduction in exendin-4-induced palmitoylation (Fig 8B), indicating that it is able to block transfer of palmitate to the GLP-1R by binding to its known palmitoylation site. We speculated that some effects of this allosteric modulator might relate to alterations in receptor conformation through chemical modification of cysteine 438, in a manner analogous to the effect of palmitoylation at this residue. However, we found that the effect of BETP on internalization of the C438A SNAP-GLP-1R mutant stimulated by exendin-phe1 was comparable to that of wild-type receptors (Fig 8C and 8D). This is in keeping with the current understanding that the pharmacology of BETP is mediated via binding to cysteine 347 rather than 438 [55] and suggests that, despite its contribution to optimal internalization, cysteine 438 palmitoylation is not an obligatory event for GLP-1R endocytosis.

As with exendin-4, β-arrestins appear dispensable for exendin-phe1-induced GLP-1R endocytosis when potentiated by BETP, as shown using the HEK293 β-arrestin-1/2 CRISPR knockout model (Fig 8E). However, BETP-induced internalization of exendin-phe1-stimulated SNAP-GLP-1R still relies on cholesterol, as pretreatment with MβCD inhibited this process to the same extent as for exendin-4 (Fig 8F). Furthermore, TIRF microscopy analysis suggested that this internalization also proceeds via a clathrin-dependent pathway, as it involved engagement of AP2 (Fig 8G) and resulted in localization of gold-labeled SNAP-GLP-1Rs to CCPs (Fig 8H). Overall, these data suggest that BETP favors the same endocytic route as that followed by GLP-1Rs after orthosteric agonist stimulation.

## Discussion

The role of the plasma membrane lipid environment in the control of GPCR functional selectivity remains a relatively underexplored area, primarily because of the technical challenges associated with the study of lipid–protein cross talk [7]. Despite this, emerging evidence using newly developed methods places lipid nanodomains as key regulators of GPCR signaling [2,5,63,66,67].

In the present study, we have unveiled a central role for this lipid context in the modulation of the biological action of the beta cell GLP-1R, a class B GPCR with a significant role in metabolic regulation and a prime T2D target [25]. We found that GLP-1R clustering within beta cell membrane nanodomains and receptor palmitoylation at its C-terminal end are agonist-regulated processes, indicating that the GLP-1R interaction with its surrounding lipid environment is conditioned by its activation state. These processes are likely to be interconnected. Agonist-induced changes to GPCR conformation, typically involving outward movement of transmembrane (TM) helix 6, may result in energetically unfavorable “hydrophobic mismatch” between the exterior receptor surface and local membrane structures [2]. Consequently, the activated receptor may seek out membrane nanodomains with altered lipid content and hydrophobicity, and receptor oligomer assembly may be favored through stable interactions between complementary hydrophobic TM helix interfaces. Accordingly, our data show that disruption of membrane lipid organization through cholesterol-depleting MβCD treatment leads to reductions in agonist-induced GLP-1R clustering. Similarly, we found that the palmitoylation-deficient GLP-1R C438A mutant was less likely to be found in membrane rafts when activated. Palmitoylation is widely considered a dynamic raft-targeting mechanism for GPCRs, as the attached acyl chains anchor the receptor via its C-terminus in lipid-rich membrane regions [17,68]. Notably, membrane-localized palmitoyl acyltransferase enzymes and protein thioesterases can themselves be palmitoylated [69] and might therefore be preferentially situated in rafts. With this in mind, it is plausible that receptor oligomerization, clustering, and palmitoylation could be mutually augmented, which could explain why residual exendin-4-induced clustering was observed both after MβCD treatment and with the GLP-1R C438A palmitoylation-null mutant. Nevertheless, there are many examples of palmitoylated proteins that are not raft-associated [69], including the commonly used non–raft marker transferrin receptor, and raft partitioning of certain palmitoylated proteins such as caveolin-1 is not affected after removal of its palmitoylation sites by mutagenesis [10], highlighting the multifactorial and complex nature of this process.

Modulation of GPCR posttranslational modifications, as well as receptor clustering and recruitment to specific plasma membrane nanodomains, have all previously been suggested as means by which biased signaling can be regulated [58]. We found marked differences in GLP-1R clustering and nanodomain recruitment between biased exendin-4-based agonists previously shown by us to exhibit significant differences in receptor binding affinity and signaling preference [22], as well as distinct GLP-1R palmitoylation profiles as shown in this study. Related to this, our single-molecule tracking analysis also demonstrates that the plasma membrane lateral diffusion of GLP-1R molecules is altered upon agonist binding, with high-affinity agonists decreasing the average diffusion coefficient of the receptor, as recently reported for the closely related class B GPCR glucagon receptor (GCGR) [70], whereas the lower-affinity biased agonist exendin-phe1 causes virtually no change. Although we did not determine the structural basis for these distinct behaviors, it seems plausible that specific TM helix rearrangements stabilized by different agonists, as previously reported for the GLP-1R [71], could affect how the receptor interacts with other membrane constituents via phenomena discussed in the previous paragraph.

The observation that these effects are recapitulated to a certain extent by the palmitoylation-null C438A GLP-1R mutant suggests a partial role for this posttranslational modification in the establishment of signal bias. However, the observed reductions in receptor clustering, raft segregation, and signaling, as well as the reduction in β-arrestin recruitment and GLP-1R internalization, were milder with the latter compared to exendin-phe1. Additionally, allosteric augmentation of agonist residence time with the GLP-1R-specific PAM BETP is in itself capable of inducing nanodomain clustering. BETP is able, as shown here, of exerting its effects while at the same time inhibiting GLP-1R palmitoylation, the latter presumably because of its covalent binding to GLP-1R cysteine 438 [55], which would block any posttranslational modification at this residue. Whether binding of BETP at cysteine 438 or at other alternative cysteine sites could functionally substitute for the absence of palmitic acid at this residue to promote nanodomain translocation was not formally assessed in the present study, but it appears improbable that the mechanism is C438-specific, as the effect of BETP on GLP-1R endocytosis was preserved in the GLP-1R C438A mutant, which was also previously reported to show normal potentiation of partial agonist signaling responses in contrast to C347A [55]. Taken together, the above considerations suggest that the control of GLP-1R recruitment to membrane nanodomains and its associated effects on receptor signaling are likely to encompass contributions from various factors.

As well as receptor conformational changes influencing interactions with plasma membrane lipids, the converse can also be true. For example, binding to a specific raft lipid has been shown to allosterically change the conformation of the human epidermal growth factor (EGF) receptor [72], and interactions with cholesterol can increase the binding affinity of certain GPCRs [73], with molecular dynamics simulations suggesting that cholesterol allosterically modulates receptors by limiting their conformational flexibility [74]. Thus, functional association of GLP-1R with membrane rafts might potentially induce specific conformational changes that could modulate its agonist binding properties. In this context, receptor clustering associated with nanodomain segregation might dynamically influence ongoing agonist binding events. Firstly, cross talk within oligomeric complexes is known to alter the binding properties of individual receptor protomers [75], although this typically manifests as negative cooperativity, i.e., with secondary protomers displaying reduced affinity, as previously described for GLP-1R constitutive homodimers [37]. Alternatively, aggregation of receptors within specific membrane regions could shift the behavior of a dissociating agonist toward rebinding to a nearby available receptor rather than diffusing away into the extracellular space [76]. Apparent support for the latter phenomenon was provided by our cholesterol depletion studies in exendin-4-stimulated conditions, in which an association was seen between reduced GLP-1R clustering and reduced binding affinity for exendin-4. However, examining this further using two agonists (exendin-4 and exendin-phe1) with opposing clustering capabilities and bidirectional manipulation of this process with BETP and MβCD provided mixed results. Specifically, the former selectively enhanced exendin-phe1 binding, whereas the effect of the latter was equal for both agonists. The likelihood that these manipulations affect multiple nonoverlapping cellular processes beyond their effects on receptor clustering, potentially including direct allosteric control of binding events by cholesterol [77], may underlie these discrepancies.

Membrane nanodomains are thought to compartmentalize cellular processes by contributing to the organization of signaling molecules. Our observation that the main GLP-1R signal transducer, GαS, is predominantly raft-associated in beta cells underlines the importance of agonist-induced GLP-1R nanodomain segregation in the optimization of GLP-1R signaling, with disturbances in this process predicted to carry deep consequences for the control of insulin secretion, as illustrated in the present study by the acute signaling and insulin secretion defects harbored by the C438A palmitoylation-null mutant. Interestingly, the same agonist dependency for either palmitoylation or membrane raft recruitment was not found with the closely related GIPR, which has a higher constitutive activity in the absence of agonist stimulation [78]. Although we did not examine this possibility, local control of cAMP and PKA signaling might involve A-kinase anchoring proteins (AKAPs) [79]. For example, the AKAP79/150 signaling complex [80], known to be palmitoylated and targeted to lipid raft domains [81] and therefore likely to be in close apposition to activated GLP-1Rs, increases GLP-1-induced insulin secretion through anchoring of multifunctional protein phosphatases [82].

Interestingly, despite being recruited to lipid rafts in response to agonist stimulation, the GLP-1R appears to follow a clathrin-dependent pathway of internalization. Additionally, we found that presence of cholesterol was required to ensure efficient GLP-1R endocytosis, as demonstrated by the inhibition of agonist-induced GLP-1R internalization following cholesterol extraction with MβCD. This phenotype correlated with concomitant reductions in agonist binding affinity and GLP-1R clustering upon MβCD exposure, with all these effects triggered at similar MβCD concentrations. Cholesterol extraction is known to inhibit both clathrin-dependent [83,84] and clathrin-independent [85] pathways of endocytosis, as, to varying extents, all endocytosis pathways are sensitive to cholesterol depletion, with some of them also being interconnected [86,87]. It can therefore be technically challenging to distinguish between endocytic pathways and their functional reliance on membrane nanodomains, with cholesterol dependency not necessarily an adequate tool to do so [88]. Moreover, the same raft‐associated protein can occasionally be internalized via different pathways [85], and blocking one pathway with either a specific chemical inhibitor or using a genetic approach such as small interfering RNA (siRNA)-mediated depletion of specific endocytic factors can have indirect effects and/or result in the up-regulation of residual pathways as a compensatory mechanism [89]. Although our direct observation of the association of clustered GLP-1Rs with the clathrin adaptor AP2 as well as presence of gold-labeled GLP-1Rs in CCPs points toward clathrin-mediated endocytosis as the main GLP-1R internalization pathway in beta cells under normal conditions, it remains to be established whether the receptor can follow alternative pathways under different contexts and/or cell types.

It is also not yet known how the receptor internalizes from cholesterol-rich membrane nanodomains. This has previously been described for other raft-associated GPCRs as either following an atypical clathrin-mediated mechanism [48] or via a sequential association to membrane rafts and CCPs [90], with raft recruitment preceding CCP incorporation. Additionally, although recruitment of β-arrestins to the GLP-1R occurred preferentially at membrane rafts, indicative of a higher level of receptor activation within these nanodomains and presumably associated with the β-arrestin-dependent GLP-1R desensitization previously observed by us [22], β-arrestins appeared to play a very minor role in GLP-1R endocytosis. It is worth noting that the GLP-1R harbors an AP2-binding domain in its C-terminal tail and can interact directly with this clathrin adaptor, reducing the need for additional intermediaries [91].

In conclusion, in the present study, we have employed biochemical assays in conjunction with high-resolution microscopy and spatially localized FRET biosensor experiments to establish the existence of an agonist-triggered GLP-1R palmitoylation and clustering mechanism coupled to nanodomain-dependent GLP-1R signaling and internalization. We have also established that this mechanism can be controlled by the modulation of agonist binding affinities, either with biased GLP-1R agonists or with the PAM BETP. Future investigations—including identification of the specific palmitoyltransferase(s) and palmitoyl protein thioesterase(s) responsible for the regulation of agonist-induced GLP-1R palmitoylation in beta cells, as well as use of molecular dynamics simulations, model systems to fine-tune the plasma membrane lipid composition, and superresolution experiments to examine dynamic changes in GLP-1R conformation and binding affinities associated with interactions with specific lipid nanodomains at a single-molecule level in real time [5,92]—will all be required to further develop our understanding of the impact of membrane lipid composition on the spatiotemporal organization of GLP-1R signaling.

## Materials and methods

### Ligands

GLP-1(7–36)NH_2_, exendin-4, and exendin(9–39) were from Bachem, and custom peptides were from Insight Biotechnology. TMR and FITC conjugates of exendin-4 and/or exendin-phe1 and exendin(9–39) were coupled via K12, as previously described [22]. BETP was from Sigma-Aldrich.

### Cell culture and generation of stable cell lines

HEK293 cells (ECACC) were maintained in DMEM supplemented with 10% fetal bovine serum (FBS) and 1% penicillin/streptomycin. CHO-K1 cells (ECACC) were maintained in DMEM supplemented with 10% FBS, 20 mM HEPES, 1% nonessential amino acids, and 1% penicillin/streptomycin. PathHunter CHO-GLP-1R β-arrestin-2 reporter cells (DiscoverX) were maintained in the manufacturer’s Culture Medium 6. HEK293 β-arrestin-2-EA cells were generated by infection of HEK293 cells with viral retroparticles expressing β-arrestin-2 fused to EA (DiscoverX) and selected in hygromycin. Parental INS-1 832/3 cells and INS-1 832/3 cells with endogenous GLP-1R or GIPR deleted by CRISPR/Cas9 (a gift from Dr. Jacqui Naylor, MedImmune) [40] were maintained in RPMI-1640 at 11 mM D-glucose, supplemented with 10% FBS, 10 mM HEPES, 2 mM L-glutamine, 1 mM pyruvate, 50 μM β-mercaptoethanol, and 1% penicillin/streptomycin. MIN6B1 cells (a gift from Prof. Philippe Halban, University of Geneva, Switzerland) were maintained in DMEM at 25 mM D-glucose supplemented with 15% FBS, 50 μM β-mercaptoethanol, and 1% penicillin/streptomycin. MIN6B1 cells with deleted endogenous GLP-1R expression were generated by CRISPR/Cas9 as follows: cells were cotransfected with the CRIPSR/Cas9 vector pX330-U6-Chimeric_BB-CBh-hSpCas9 (a gift from Prof. Feng Zhang, Addgene plasmid #42230) with cloned guide RNA sequence 5′-CCCCGAGCAGCAGGAGCGCC-3′ targeting the minus strand of mouse GLP-1R exon 1 and pcDNA3.1+ and selected in 1 mg/ml G418. A mixed population of cells was recovered and cultured without further G418 selection. Stable SNAP-GLP-1R-expressing HEK293 cells (wild type and β-arrestin-less [52]) were generated by transfection of pSNAP-GLP-1R (Cisbio) and G418 (1 mg/ml) selection. The same approach was used to generate HEK293 cells stably expressing N-terminal FLAG-tagged GLP-1R and to reintroduce stable expression of wild-type or C438A mutant SNAP-GLP-1R into INS-1 832/3 cells lacking endogenous GLP-1R expression. Stable SNAP-GLP-1R CHO-K1 and MIN6B1 cells were described previously [22]. Mycoplasma testing was performed yearly.

### Generation of SNAP-GLP-1R C438A and PK-tagged SNAP-GLP-1R wild-type and C438A vectors

SNAP-GLP-1R C438A mutant vector was generated from wild-type SNAP-tagged human GLP-1R by site-directed mutagenesis with the QuikChange II XL Site-Directed Mutagenesis Kit (Agilent), following the manufacturer’s instructions. PK-tagged variants of both SNAP-GLP-1R wild-type and C438A vectors were generated for β-arrestin-2 recruitment assays by cloning of HindIII–BglII restriction fragments onto the pCMV-ProLink 1 Vector (DiscoverX).

### MβCD treatments

Cells were treated with MβCD (Sigma-Aldrich) at the indicated concentration in HBSS or media without serum. MβCD treatments were washed off before stimulation, with the exception of the time-lapse confocal microscopy and the TR-FRET assays performed with multiple MβCD concentrations, in which for practical reasons, MβCD was left on the cells during agonist treatment. When relevant, MβCD treatments were performed after labeling with SNAP-Surface fluorescent probes.

### Fluorescent labeling and biochemical quantification of cellular cholesterol

Cells were treated with or without MβCD as above. For cholesterol labeling, filipin staining was carried out with the Cell-Based Cholesterol Assay Kit (Abcam). For quantification of cholesterol levels, lipid was extracted using butanol as described [93]. Briefly, the washed cell pellet was left in 250 μl butanol overnight at 4 °C, and the organic layer was aspirated, placed in a microplate, and allowed to evaporate to dryness. The lipid film was redissolved in 1% Triton X-100, and cholesterol concentration was determined using the Amplex Red Cholesterol Assay Kit (Thermo Fisher), followed by normalization to protein content of the cell pellet determined by BCA assay.

### Cell labeling for EM

SNAP-GLP-1R-expressing cells cultured on Thermanox coverslips (Agar Scientific) were labeled with 2 μM cleavable SNAP-Surface biotin probe in full media, followed by 5 μg/ml NaN_3_-free Alexa Fluor 488 Streptavidin, 10 nm colloidal gold conjugate (Thermo Fisher) in HEPES-bicarbonate buffer (120 mM NaCl, 4.8 mM KCl, 24 mM NaHCO_3_, 0.5 mM Na_2_HPO_4_, 5 mM HEPES, 2.5 mM CaCl_2_, and 1.2 mM MgCl_2_, saturated with 95% O_2_/5% CO_2_ [pH 7.4]), and 1% BSA and stimulated with the indicated treatment. Conventional EM was performed as described. Briefly, cells were fixed, processed, mounted on Epon stubs, and polymerized at 60 °C, and 70-nm sections were cut en face with a diamond knife (DiATOME) in a Leica Ultracut UCT ultramicrotome before examination on an FEI Tecnai G2-Spirit TEM. Images were acquired in a charge-coupled device camera (Eagle) and processed in Fiji.

### EM quantification of clustering by plasma membrane rip-offs

Cells expressing SNAP-GLP-1R were cultured on Thermanox coverslips and gold-labeled with SNAP-Surface biotin followed by AlexaFluor 488 Streptavidin, 10 nm colloidal gold conjugate as above. Cells were stimulated with the indicated treatments for 1 min. Membrane rip-offs were performed following the protocol of Sanan and Anderson [94]. In brief, EM grids were coated with formvar and poly-L-lysine, and cell coverslips were inverted on top with pressure applied for approximately 10 s at 4 °C. This allowed detachment of the apical cellular membranes onto the grids, which were immediately fixed by incubating in 4% glutaraldehyde in 25 mM HEPES buffer for 15 min. The grids were subsequently prepared for EM analysis by postfixation in 2% aqueous osmium followed by 1% aqueous tannic acid and 1% aqueous uranyl acetate, for 10 min each at room temperature, and examined on a JEOL 1400+ TEM with images taken on an AMT digital camera. Gold-particle nearest-neighbor analysis was performed using ImageJ.

### Cell labeling, confocal, and TIRF microscopy

For confocal microscopy, cells were labeled at 37 °C with 1 μM of the indicated SNAP-tag fluorescent probe (New England Biolabs) in full media, stimulated with agonists for the indicated times, fixed in 4% paraformaldehyde, mounted in Prolong Diamond antifade reagent with 4,6-diamidino-2-phenylindole (Life Technologies), imaged with a Zeiss LSM-780 inverted confocal laser-scanning microscope in a ×63/1.4 numerical aperture oil-immersion objective from the Facility for Imaging by Light Microscopy (FILM) at Imperial College London, and analyzed in Fiji. For TIRF microscopy, SNAP-GLP-1R-expressing cells were transfected with μ2-HA-WT (a gift from Prof. Alexander Sorkin, Addgene plasmid #32752), plated on glass-bottom MatTek dishes, labeled with SNAP-Surface 488 probe as above, stimulated or not for 2 min with the indicated agonist, and fixed as above before immunofluorescence with a mouse monoclonal anti-HA (HA-7) antibody (catalogue no. H3663, Sigma), followed by a secondary AlexaFluor 546 antibody. Unmounted samples in PBS were imaged using a Nikon Eclipse Ti microscope equipped with a ×100/1.49 numerical aperture TIRF objective, a TIRF iLas2 module, and a Quad Band TIRF filter cube (TRF89902, Chroma). Images were acquired with an ORCA-Flash 4.0 camera (Hamamatsu) and Metamorph software (Molecular Devices), and colocalization was measured by Manders’ coefficient analyzed using Coloc 2 plugin in Fiji.

### Analysis of SNAP-GLP-1R internalization and fluorescent exendin-4 conjugate uptake by time-lapse confocal microscopy

To assess dynamic internalization profiles of the SNAP-GLP-1R, cells were plated onto glass-bottom MatTek dishes and labeled with SNAP-Surface 549 prior to imaging using the same confocal system as above in HBSS at 37 °C for 10 min at 0.33 frames s^−1^ immediately after stimulation with 100 nM exendin-4. For ligand uptake measurements, unlabeled cells were stimulated with 100 nM exendin-4-K12-TMR. Levels of surface SNAP-GLP-1Rs or exendin-4-K12-TMR were analyzed using a custom algorithm. Briefly, intensity profiles were obtained from three separate segments across the plasma membrane of individual cells for every 10th frame. Peak intensities were calculated from these segments and averaged. Data was normalized to baseline at time 0 for each individual acquisition and plotted over time. Area under the curve (AUC) was calculated from kinetic traces to compare internalization propensities.

### Clustering measurements by TIRF-PALM

For TIRF-PALM analysis of FLAG-GLP-1R cluster sizes, the GLP1R-Tango vector (a gift from Prof. Bryan Roth, Addgene plasmid #66295), which harbors a single N-terminal FLAG tag, was used to introduce a STOP codon at the end of the GLP-1R coding sequence by site-directed mutagenesis performed as above. HEK293 cells were used to generate a subline with stable expression of the engineered FLAG-GLP-1R construct as above. Cells were subsequently plated onto glass-bottom MatTek dishes and labeled with the anti-FLAG GAGE 500 antibody (1:500 dilution) described in [95] in 10% FCS in PBS containing calcium and magnesium at 37 °C for 30 min. Cells were washed in PBS containing calcium and magnesium and stimulated or not with 100 nM exendin-4 for 2 min prior to fixation for 30 min in 4% paraformaldehyde with 0.2% glutaraldehyde to minimize antibody-induced clustering artifacts. Following fixation, cells were washed in PBS with calcium and magnesium and maintained in the same buffer for imaging. All steps were carried out in the dark to ensure minimal label photo-switching. Images were acquired using a Zeiss Elyra PS1 featuring an AxioObserver Z1 motorized inverted microscope with TIRF capability with an Alpha Plan-APO ×100/1.46 numerical aperture oil-immersion objective at the Imperial College London FILM facility. Photo-conversion of CAGE 500 was achieved with a 405-nm light source and simultaneously imaged and photo-bleached with a 488-nm laser line. The microscope was contained in a plastic draft-proof enclosure maintained at a constant temperature of 25 °C and mounted on a vibration isolation table. Laser lines were switched on at least 1 h prior to imaging to allow stabilization of the system in order to ensure minimal sample drift throughout the imaging. Each TIRF-PALM time series was acquired using a cooled electron-multiplying charge-coupled device camera (EM-CCD; iXon DU 897, Andor Technology) and LMS Zen operating software with an exposure time of 30 ms. Localization of receptors was determined using QuickPALM Fiji plugin: fluorescent images of cropped nonoverlapping areas of 7 × 7 μm within cell borders were analyzed using a full-width half-maximum value of 4 and signal-to-noise ratio of 8. Analyzed areas did not span cell membranes, to exclude any potential biasing resulting from edge effects. Data tables containing particle-localization 2D coordinates were generated. The number of associated receptor molecules from these coordinates was determined with PD-Interpreter as in [95] with a 50-nm radius from each fluorophore. To discount any overestimation of oligomers, events within a 10-nm radius of an activated fluorophore were discounted from the analysis.

### Single-molecule localization microscopy

CHO-K1 cells were cultured in phenol red-free DMEM/F12, supplemented with 10% FBS at 37 °C with 5% CO_2_ and seeded onto 25-mm clean glass coverslips at a density of 3 × 10^5^ cells per well. The following day, cells were transfected with SNAP-GLP-1R, labeled 4 h after transfection with 1 μM SNAP-Surface 549 for 20 min in complete medium, washed and imaged in culture medium at 37 °C using a custom-built TIRF microscope (Cairn Research) based on an Eclipse Ti2 (Nikon, Japan) equipped with four EMCCD cameras (iXon Ultra, Andor), a 561-nm diode laser, and a ×100/1.49 numerical aperture oil-immersion objective (Nikon). Cells were stimulated with either vehicle or 100 nM exendin-4, exendin-phe1, or exendin-asp3 for 10–15 min. Single-molecule image sequences were acquired every 30 ms. Image sequences were analyzed with an automated particle detection and tracking algorithm (u-track) [59] in the MATLAB environment and further investigated using custom algorithms, as previously described [59,60]. To analyze the motion of receptors, the time-averaged MSD (TA-MSD) [96] of individual trajectories from TIRF image sequences was computed as previously described [60], and fitted with the following equation:

$$\text{TA-MSD}\left(\text{t}\right)=4{\text{Dt}}^{\alpha }+4\sigma_{\text{err}}{}^{2}$$

where t indicates time, α is the anomalous diffusion exponent, and *σ*_err_ is a constant offset for localization error. Only trajectories lasting at least 100 frames were analyzed and categorized according to the diffusion parameters D and α. Particles with D < 0.01 μm^2^ s^−α^ were considered immobile. Normal diffusion was assigned to particles with D ≥ 0.01 μm^2^ s^−α^ and 0.75 ≤ α ≤ 1.25. Sub- and superdiffusion were assigned to particles with D ≥ 0.01 μm^2^ s^−α^ and α < 0.75 or α > 1.25, respectively.

### Clustering measurements by TR-FRET

Cells stably or transiently expressing SNAP-GLP-1R were dual-labeled with the TR-FRET probe SNAP-Lumi4-Tb (40 nM, Cisbio) and SNAP-Surface 647 (1 μM, New England Biolabs) for 60 min at room temperature. These concentrations were selected because they provided optimum signal intensity at both measurement wavelengths. Where relevant, MβCD treatments were performed after labeling. Cells were washed and placed in HBSS in a white plate for a 10-min baseline measurement at 37 °C using a Spectramax i3x (Molecular Devices) plate reader fitted with a TR-FRET filter set (λEx = 335 nm, λEm = 616 nm and 665 nm, delay 30 μs, integration time 400 μs). TR-FRET was sequentially measured after agonist addition. The ratio of fluorescent signals at both emission wavelengths (665/616) was considered indicative of clustering because it reflects both transient and stable interactions between receptor protomers occurring within the long fluorescence lifetime of the excited terbium cryptate. Dose-response curves were constructed from AUC obtained from kinetic traces at different agonist concentrations and fitted to 4-parameter logistic curves to calculate potency (log EC_50_) and efficacy (E_max_) values.

### NR12S assays and analysis

Cells expressing SNAP-GLP-1R were first labeled with SNAP-Lumi4-Tb (40 nM) for 60 min at room temperature. Where relevant, MβCD treatments were performed after Lumi4-Tb labeling. For spectral measurements and analysis, (1) washed, Lumi4-Tb-labeled cells were placed in HBSS in a white plate, and a full baseline TR-FRET spectral scan was performed at 37 °C using a Flexstation 3 plate reader (Molecular Devices; λEx = 335 nm, λEm = 460–650 nm in 5-nm steps, no cutoff, delay 50 μs, integration time 300 μs) to detect the Lumi4-Tb-only signal; (2) freshly prepared NR12S in HBSS (200 nM) was then added, and after a further 5 min, a second spectral scan was performed to detect basal FRET; (3) agonist was added for 5 min, and a final spectral scan was performed to detect agonist-changes in TR-FRET. After an initial blank subtraction, data were analyzed by ratiometrically normalizing the spectrum from each read to the signal measured at 490 nm; the normalized Lumi4-Tb-only read was subtracted from subsequent normalized reads to obtain the NR12S-specific TR-FRET signal in the presence and absence of agonist. Where indicated, the AUC was calculated below and above the λEm maximum of 590 nm as a marker of relative contributions from liquid-ordered-associated (530–590 nm) and liquid-disordered-associated (590–650 nm) SNAP-GLP-1Rs and expressed relative to the total NR12S TR-FRET AUC (530–650 nm). Alternatively, liquid-ordered and liquid-disordered FRETs were assigned values of 570 and 610 nm, respectively, with the NR12S-associated increase at each wavelength ratiometrically expressed to indicate differences in SNAP-GLP-1R localization in each nanodomain. For kinetic measurements and analysis, washed, Lumi4-Tb-labeled cells were treated with NR12S at the indicated concentration, and a 5-min baseline read was performed (λEx = 335 nm, λEm = 490, 570, and 610 nm). Agonist was then added and changes in TR-FRET sequentially monitored. Signals at each wavelength were blank-subtracted and expressed ratiometrically to each other, with agonist-induced changes quantified relative to individual well baseline.

### Measurement of GLP-1R binding parameters by TR-FRET

For equilibrium binding assay at multiple MβCD concentrations, cells expressing SNAP-GLP-1R were first labeled with SNAP-Lumi4-Tb (40 nM) for 60 min at room temperature and added to 96-well white plates. MβCD treatments in HBSS were applied for 45 min along with metabolic inhibitors (20 mM 2-deoxyglucose and 10 mM NaN_3_) to prevent GLP-1R endocytosis [97]. Exendin-4-K12-FITC or exendin-9-K12-FITC at a final BSA concentration of 0.1% were applied and allowed to bind at room temperature for 3 h. TR-FRET was then measured over 10 min at room temperature using a Flexstation 3 plate reader (λEx = 335 nm, λEm = 520 and 620 nm, delay 50 μs, integration time 300 μs). Binding was quantified ratiometrically as a 520/620 signal, and K_d_ was determined using the Prism saturation binding model “one site–specific binding”. For kinetic binding assays, cells expressing SNAP-GLP-1R were first labeled with SNAP-Lumi4-Tb (40 nM) for 60 min at room temperature. Where relevant, MβCD treatments were performed after Lumi4-Tb labeling. After washing, cells were placed in HBSS + 0.1% BSA, supplemented with metabolic inhibitors as above, and baseline TR-FRET was measured over 10 min at 37 °C as above. FITC-conjugated agonists were then added, and TR-FRET was sequentially measured to monitor agonist association. Binding was quantified ratiometrically as a 520/620 signal, and baseline-subtracted data were analyzed using the GraphPad Prism model “association kinetics: two or more concentrations of hot” to obtain association (*k*_on_) and dissociation (*k*_off_) rate constants, from which the equilibrium binding constant K_d_ was determined.

### Measurement of GLP-1R internalization by DERET assay

The assay was performed as previously described [22]. Cells expressing SNAP-GLP-1R were first labeled with SNAP-Lumi4-Tb (40 nM) for 1 h at room temperature. Where relevant, MβCD treatments were performed after Lumi4-Tb labeling. After washing, cells were placed in HBSS supplemented with 24 mM fluorescein. A 10-min baseline read at 37 °C was taken in a Flexstation 3 plate reader in TR-FRET mode (λEx = 335 nm, λEm = 520 and 620 nm, delay 400 μs, integration time 1,500 μs), after which compounds were injected to the wells. Loss of surface SNAP-GLP-1R was detected as changes over time in the homogenous time-resolved fluorescence (HTRF) ratio (620/520 signal after blank subtraction at each time point). Dose-response curves were constructed from AUC obtained from kinetic traces at different agonist concentrations and fitted to 4-parameter logistic curves to calculate potency (log EC_50_) and efficacy (E_max_) values.

### Measurement of FITC-ligand uptake by TR-FRET

Cells expressing SNAP-GLP-1R were first labeled with SNAP-Lumi4-Tb (40 nM) for 60 min at room temperature. After washing, MβCD treatments in HBSS were added to the cells directly in 96-well plates for 45 min. Cells were then moved to the cold room to temporarily arrest endocytic processes before addition of 100 nM exendin-4-K12-FITC or exendin(9–39)-K12-FITC, which were allowed to bind for 3 h at 4 °C to reach a state of quasi-equilibrium. The plate was then moved directly to a Flexstation 3 plate reader at 37 °C to initiate endocytosis of prebound FITC-ligand with the extracellular ligand concentration remaining constant. TR-FRET signal was monitored as above (λEx = 335 nm, λEm = 520 and 620 nm, delay 50 μs, integration time 300 μs) over 30 min. Here, entry to the endosomal pathway leads to dissociation of labeled receptor-ligand complexes, resulting in a reduction in TR-FRET signal. The acidic endosomal pH also reduces inherent FITC fluorescence. Net ligand uptake was therefore calculated as the AUC for change in TR-FRET signal throughout the 30-min assay period.

### HTRF cAMP measurements

Cells were treated in white microplates with agonist, with or without isobutylmethylxanthine (IBMX) as indicated to inhibit phosphodiesterases, for the indicated period of time before total cellular cAMP determination by HTRF (Cisbio cAMP Dynamic 2).

### Measurement of β-arrestin-2 recruitment by PathHunter assay

Cells were treated with agonist or drug at the indicated concentration for the indicated time periods before lysis and/or membrane fractionation and detection of β-arrestin-2 recruitment by the PathHunter enzyme fragment complementation assay (DiscoverX) according to the manufacturer’s instructions.

### Real-time measurement of cAMP production and PKA activation using FRET biosensors

The ^T^Epac^VV^ biosensor was a gift from Dr. K. Jalink, The Netherlands Cancer Institute. The AKAR4-NES and AKAR4-Lyn biosensors were gifts from Dr Jin Zhang (Addgene plasmid #61620). Cells expressing SNAP-GLP-1Rs were transiently transfected with the relevant biosensor plasmid for 36 h prior to the assay and placed in HBSS in 96-well clear-bottom black plates. After a 5-min baseline read at 37 °C (λEx = 440 nm, λEm = 485 and 535 nm) in a Flexstation 3 plate reader, compounds were injected to each well, and sequential readings immediately commenced. FRET was expressed ratiometrically as signal at 535 nm divided by signal at 485 nm. Individual well responses were normalized to baseline to reduce variability. Dose responses were either analyzed by obtaining the cumulative AUC relative to baseline over a fixed time period, or at each time point, or time bin, to allow kinetic comparisons of agonists between each pathway. To determine agonist-related biased signaling between each biosensor, the relative potency ratio approach was taken, as all compounds were full agonists in each pathway measured [98]. Dose-response curves were constructed for each agonist at indicated time points, and log EC_50_ values for exendin-phe1 and exendin-asp3 responses in each pathway were subtracted from those of reference agonist exendin-4 to generate normalized potency estimates (ΔLog EC_50_), followed by subtraction of the test pathway (AKAR4-Lyn) from the reference pathway (AKAR4-NES or ^T^Epac^VV^) to provide the bias estimate (ΔΔLog EC_50_). As all agonists in each pathway were measured in parallel during the same experiment using the same stock of serially diluted ligand, experiment-specific ΔLog EC_50_ and ΔΔLog EC_50_ values could be produced without requiring error propagation [22].

### Insulin secretion assays

INS-1 832/3 cells with endogenous GLP-1R expression deleted by CRISPR/Cas9 (INS-1 832/3 GLP-1R^−/−^) stably expressing wild-type or C438A SNAP-GLP-1Rs were preincubated overnight in 3 mM glucose medium. Exendin-4 was added in complete medium at 11 mM glucose at the time of seeding into plates. After 16 h of incubation, samples were obtained for secreted insulin and analyzed by HTRF (Cisbio). Results were normalized as a fold increase compared to cells treated with 11 mM glucose alone.

### Palmitoylation assays

For the detection of palmitoylated GLP-1Rs, cells were stimulated with 100 nM of the indicated agonist for 10 min and, when appropriate, pretreated with 10 μM BETP for 5 min (and throughout the agonist stimulation period) or overnight with 200 μM of the palmitoylation inhibitor 2-BP. Isolation of palmitoylated proteins was performed with the CAPTUREome S-Palmitoylated Protein Kit (Badrilla), following the manufacturer’s instructions. The assay is based on the acyl-resin assisted capture method, described in detail elsewhere [99]. Key steps include blocking of free thiol groups with a blocking reagent, cleavage of thioester linkages to release the palmitate group, and capture of newly liberated thiols with the CAPTUREome capture resin. Briefly, cells were lysed and free thiols blocked in the manufacturer’s blocking buffer at 40 °C for 4 h with constant vortexing. Proteins were then precipitated with cold acetone for 20 min at −20 °C. Following centrifugation at 16,000*g* for 5 min, the pellet was extensively washed with 70% cold acetone and resuspended in the manufacturer’s binding buffer. Approximately 10% of the soluble lysate was saved as the input fraction (IF). Prewashed CAPTUREome capture resin was added to the lysates. To this mixture, either the thioester cleavage or the acyl-preservation reagents were added. Resins were washed five times with the manufacturer’s binding buffer. Urea sample buffer (100 mM Tris-HCl [pH 6.8], 2.5% SDS, 4 M urea, 50 mM dithiothreitol, 0.05% bromophenol blue) was used to elute captured proteins from the resin. Eluted samples were incubated at 37 °C for 30 min before western blotting for SNAP-GLP-1R to detect the fraction of palmitoylated versus nonpalmitoylated receptor. Resin-bound proteins treated with the thioester cleavage reagent are referred to as the cleaved bound fraction (cBF) and represent the fraction of palmitoylated proteins, whereas resin-bound proteins treated with the acyl-preservation reagent are referred to as the preserved bound fraction (pBF) and are used as negative control for the assay. Corresponding unbound fractions are known as the cleaved unbound fraction (cUF) and the preserved unbound fraction (pUF).

### Detergent-resistant and detergent-soluble membrane fractionation

For membrane raft purification experiments, the cells were seeded onto 10-cm dishes and transiently transfected with GαS-YFP (a gift from Catherine Berlot, Addgene plasmid #55781) when indicated. After the indicated treatments, cells were osmotically lysed in 20 mM Tris-HCl (pH 7.0) supplemented with cOmplete EDTA-free protease inhibitor cocktail (Roche) and phosphatase inhibitor cocktail 2 (Sigma-Aldrich). The cell suspension was homogenized by passing through a 22-gauge needle and ultracentrifuged at 63,000*g* for 1 h at 4 °C with a Sorvall Discovery M120 ultracentrifuge. The supernatant was discarded, and the pellet resuspended in ice-cold PBS supplemented with protease inhibitor cocktail and an aliquot retained for total membrane fraction (TMF) analysis. PBS was added to a final volume of 1 ml, and the suspensions were ultracentrifuged at 63,000*g* for 30 min at 4 °C. The supernatant was discarded, and the pellet was resuspended in ice-cold PBS supplemented with 1% Triton-X100 and protease inhibitor cocktail and incubated for 30 min at 4 °C. Samples were ultracentrifuged again at 63,000*g* for 1 h at 4 °C. The supernatant, containing the DSF, was retained for analysis, and the DRF pellet was resuspended in 1% SDS with protease inhibitor cocktail. All the samples were sonicated with the Ultrasonic cell disruptor XL-2000 probe sonicator (Misonix) and centrifuged at 14,000*g* for 5 min at 4 °C before analysis by SDS-PAGE and western blotting. For measurement of β-arrestin-2 recruitment to different membrane regions, purified fractions from PathHunter CHO-GLP-1R cells, prepared as above after vehicle or exendin-4 treatment, were diluted in HBSS and measured using PathHunter chemiluminescent reagents.

### SDS-PAGE and western blotting

Samples were separated by SDS-PAGE on 10% gels under reducing conditions and immunoblotted onto PVDF membranes (GE Healthcare) and bands detected by enhanced chemiluminescence (GE Healthcare) onto films developed on a Xograph Compact X5 processor. Antibodies for western blotting were anti-flotillin-1 mouse monoclonal antibody, catalogue no. sc-74566, Santa Cruz Biotechnology (1:200 dilution); anti-flotillin-1 rat monoclonal antibody, catalogue no. 849802, BioLegend (1:100 dilution); anti-SNAP-tag rabbit polyclonal antibody, catalogue no. P9310S, New England Biolabs (1:500 dilution); anti-GFP mouse monoclonal antibody, catalogue no. G6795, Sigma (1:1,000 dilution); anti-human transferrin receptor mouse monoclonal antibody, catalogue no. G1/221/12, Developmental Studies Hybridoma Bank (DSHB; 0.2 μg/ml); anti-human GLP-1R mouse monoclonal antibody, catalogue no. Mab 3F52, DSHB (0.2 μg/ml); anti-mouse GLP-1R mouse monoclonal antibody, catalogue no. Mab 7F38, DSHB (0.2 μg/ml); and anti-α-tubulin mouse monoclonal antibody, catalogue no. T5168, Sigma (1:1000 dilution). All uncropped blots showing results included in the manuscript can be found in S1 Raw Images.

### Dose-response data and statistical analyses

Statistical significance was assessed using Student’s *t* test or ANOVA as indicated, with matched analyses performed where possible. Curve fitting and statistical analyses were performed using GraphPad Prism 8.0. Statistical significance was taken as *p* < 0.05. Unless indicated, values represented are the mean ± SEM. Dose-response summaries for all the relevant manuscript figures are included in S1–S7 Tables.

## Supporting information

S1 Fig Agonist-induced SNAP-GLP-1R clustering and recruitment to membrane nanodomains—Extra data. (A) Western blotting analysis of SNAP-GLP-1R in lysates from MIN6B1 SNAP-GLP-1R cells incubated overnight with the indicated amount of tunicamycin, a drug that blocks N-linked glycosylation, showing that the two predominant bands detected for SNAP-GLP-1R at approximately 75 and 65 kDa correspond to differentially glycosylated forms of the receptor. Tubulin is shown as a loading control. (B) Confocal analysis of surface and total SNAP-GLP-1R levels in MIN6B1 SNAP-GLP-1R cells following overnight incubation in vehicle or 10 μg/ml tunicamycin. Surface SNAP-GLP-1R labeled with SNAP-Surface 488, green; total SNAP-GLP-1R labeled with SNAP-Cell TMR-Star, red; nuclei (DAPI), blue; size bars, 100 μm. (C) Level of SNAP-GLP-1R in DRFs isolated from MIN6B1 SNAP-GLP-1R cells treated with vehicle, exendin-4 or GLP-1 for 2 min, with flotillin as a marker of membrane raft loading. Fold increase versus vehicle = 1.96 ± 0.11 for 1 nM exendin-4, 2.02 ± 0.11 for 10 nM exendin-4, and 2.08 ± 0.23 for 100 nM GLP-1. (D) Cartoon explaining NR12S assay to monitor SNAP-GLP-1R translocation to membrane nanodomains. (E) TR-FRET spectra in Lumi4-Tb-labeled HEK SNAP-GLP-1R cells treated sequentially with 1 μM NR12S and 100 nM exendin-4, normalized to signal at 490 nm, *n* = 3. (F) NR12S-associated TR-FRET spectra in Lumi4-labeled HEK293 SNAP-GLP-1R cells treated with the indicated concentrations of exendin-4 or vehicle, Lumi4-Tb-only spectrum has been subtracted, and spectrum divided into Lo (530–590 nm) and Ld (590–650 nm) regions, *n* = 6; error bars not shown for clarity. (G) Proportional change in Lo-associated (left) or Ld-associated (right) SNAP-GLP-1R-NR12S TR-FRET induced by exendin-4, determined from (F) as percentage of total AUC from 530–590 nm or 590–650 nm portions of the spectrum, respectively, 3-parameter fits of pooled data shown. (H) Alternative analysis of data from (F), with TR-FRET increase after Lumi4-Tb-only subtraction quantified at 570 nm and 610 nm and expressed ratiometrically to indicate increased localization of SNAP-GLP-1R in Lo phase, 3-parameter fit of pooled data shown. All data are shown as mean ± SEM except where indicated. (I) Further examples of electron micrographs showing clusters of gold-labeled SNAP-GLP-1Rs (arrows) from 2D plasma membrane sheets isolated from MIN6B1 cells stably expressing SNAP-GLP-1R following SNAP-tag gold labeling and treatment with 100 nM exendin-4 for 2 min; size bars, 100 nm. Underlying raw data for all the panels included in this figure can be found in S2 Data; uncropped blots from this figure can be found in S1 Raw Images. AUC, area under the curve; DRF, detergent-resistant fraction; GLP-1R, glucagon-like peptide-1 receptor; HEK, human embryonic kidney; Ld, liquid-disordered; Lo, liquid-ordered; TMR, 5-Carboxytetramethylrhodamine; TR-FRET, time-resolved Förster resonance energy transfer.
(EPS)

S2 Fig Effects of inhibition of nanodomain compartmentalization on GLP-1R responses—Extra data. (A) Cholesterol levels determined by filipin staining (left) in CHO SNAP-GLP-1R cells after SNAP-Surface 549 labeling (right) treated with vehicle, MβCD (10 mM), or MβCD saturated with cholesterol for 1 h as a control; size bars, 10 μm. (B) Biochemical quantification of cholesterol depletion by MβCD in HEK293, CHO-K1, INS-1 832/3, and MIN6B1 cells treated for 45 min with 10 mM MβCD (3 mM for CHO-K1 cells) followed by butanol extraction and cholesterol quantification and normalization to protein content, *n* = 3. (C) Lack of effect of MβCD (10 mM, 45 min) treatment on surface labeling by Lumi4-Tb in HEK SNAP-GLP-1R cells, measured as TR-FRET at 550 nm and normalized for cell count, *n* = 3. (D) Equilibrium binding assay showing binding of exendin-4-K12-FITC to INS-1 832/3 GLP-1R^−/−^ SNAP-GLP-1R cells treated with indicated concentration of MβCD (45 min), *n* = 5. (E, F) Binding traces (E) and corresponding association (*k*_on_) and dissociation (*k*_off_) rate constants (F) for exendin-4-K12-FITC determined from TR-FRET kinetic binding experiments in INS-1 832/3 GLP-1R^−/−^ SNAP-GLP-1R cells with or without prior cholesterol depletion with MβCD (10 mM, 45 min), *n* = 5, paired *t* test. (G) SNAP-GLP-1R clustering responses at indicated dose of exendin-4 in INS-1 832/3 GLP-1R^−/−^ SNAP-GLP-1R cells with and without prior treatment with MβCD (10 mM, 45 min), expressed as fold increase from baseline, *n* = 5. (H) Dose-response analysis of MβCD effect on exendin-4-induced liquid-ordered-associated SNAP-GLP-1R NR12S TR-FRET, calculated as percentage of total AUC from 530–590 nm portion of spectrum, 3-parameter fit of pooled data shown. (I) Dose responses for exendin-4-induced ^T^Epac^VV^ and AKAR4-Lyn FRET changes, determined as AUC over 30 min relative to individual baselines, paired *t* test used to compare E_max_ from *n* = 5 repeats. (J) Confocal analysis of SNAP-GLP-1R internalization in CHO SNAP-GLP-1R cells labeled with SNAP-Surface 549 prior to treatment with MβCD (10 mM) or MβCD saturated with cholesterol (as a control) for 1 h followed by 15-min stimulation with 100 nM exendin-4; size bars, 10 μm. (K) Time-course internalization, assessed as decrease in plasma membrane (surface) signal, from time-lapse confocal microscopy data of CHO SNAP-GLP-1R cells labeled with SNAP-Surface 549 for 30 min and treated or not with 10 mM MβCD for 1 h before stimulation with 100 nM exendin-4. Data normalized to baseline for every individual trace, *n* = 3; inset, AUC calculated from main graph with unpaired *t* test performed. (L) Uptake of exendin-4-K12-FITC or exendin(9–39)-K12-FITC in INS-1 832/3 GLP-1R^−/−^ SNAP-GLP-1R cells pretreated with indicated concentration of MβCD, measured by TR-FRET, shown normalized to baseline signal, *n* = 5. (M) Time-course exendin-4-K12-TMR uptake, assessed as decrease in surface signal from time-lapse confocal microscopy data of INS-1 832/3 GLP-1R^−/−^ SNAP-GLP-1R cells pretreated with the indicated MβCD concentration for 1 h. Data normalized to baseline for every individual trace, *n* = 6 traces from three time-lapse recordings per condition. *p < 0.05, **p < 0.01, “ns” indicates nonsignificant, by statistical test indicated in the text. Data are shown as mean ± SEM, with individual replicates shown where relevant. Underlying raw data for all the panels included in this figure can be found in S2 Data, and a dose-response summary for this figure is included in S6 Table. AUC, area under the curve; CHO, Chinese hamster ovary; FITC, fluorescein isothiocyanate; GLP-1R, glucagon-like peptide-1 receptor; MβCD, methyl-β-cyclodextrin; TMR, 5-Carboxytetramethylrhodamine; TR-FRET, time-resolved Förster resonance energy transfer.
(EPS)

S3 Fig Lack of effect of β-arrestins on exendin-4 binding affinity to the GLP-1R. (A) TR-FRET kinetic binding data for exendin-4-K12-FITC in wt and βarr1/2 KO cells stably expressing SNAP-GLP-1R, *n* = 4. (G) Association rate (*k*_on_), dissociation rate (*k*_off_), and equilibrium dissociation constants (K_d_) derived from (A), compared by paired *t* test, “ns” indicates nonsignificant. Data are shown as mean ± SEM, with individual replicates shown where relevant. Underlying raw data for all the panels included in this figure can be found in S2 Data. βarr1/2 KO, β-arrestin-1/2 knockout; FITC, fluorescein isothiocyanate; GLP-1R, glucagon-like peptide-1 receptor; TR-FRET, time-resolved Förster resonance energy transfer; wt, wild-type.
(EPS)

S4 Fig Nanodomain segregation, signaling, and internalization of GLP-1R C438A mutant—Extra data. (A) Stably expressed SNAP-GLP-1R wt or C438A surface levels (right) and representative images (left) in INS1 832/3 GLP-1R^−/−^ cells. SNAP-Surface 488, green; nuclei (DAPI), blue; size bars, 10 μm. Paired *t* test. (B) Surface Lumi4-Tb labeling in HEK293 cells transiently transfected with wt or C438A SNAP-GLP-1R, *n* = 9, paired *t* test. (C) TR-FRET kinetic binding data for exendin-4-K12-FITC in INS1 832/3 GLP-1R^−/−^ cells stably expressing wild-type or C438A SNAP-GLP-1R, *n* = 4. (D) Association (*k*_on_) and dissociation (*k*_off_) rate constants derived from (C), paired *t* tests. (E) Wt or C438A SNAP-GLP-1R clustering responses at indicated dose of exendin-4 in INS-1 832/3 GLP-1R^−/−^ SNAP-GLP-1R cells, measured by HTRF and expressed as fold increase from baseline, *n* = 5. (F) Endogenous level of GLP-1R expression in lysates from MIN6B1 wt versus GLP-1R^−/−^ cells. Tubulin is shown as a loading control. (G) Confocal analysis of SNAP-GLP-1R wt versus C438A internalization in MIN6B1 GLP-1R^−/−^ cells transiently expressing either type of SNAP-GLP-1R following labeling with SNAP-Surface 488 for 30 min and stimulation with 100 nM exendin-4 for 15 min. Nuclei (DAPI), blue; size bars, 10 μm. (H, I) Confocal analysis of SNAP-GLP-1R wt versus C438A plasma membrane recycling in INS-1 832/3 GLP-1R^−/−^ (H) and MIN6B1 GLP-1R^−/−^ (I) cells expressing either wt or C438A mutant SNAP-GLP-1R. Cells were stimulated with 100 nM exendin-4 for 1 h to allow for maximal internalization of the receptor, followed by washout and a further 3-h incubation with 10 μM of the GLP-1R antagonist exendin(9–39) to allow for recycled receptors to accumulate at the plasma membrane. Nuclei (DAPI), blue; size bars, 10 μm. (J) Wt or C438A SNAP-GLP-1R internalization responses at indicated dose of exendin-4 in INS-1 832/3 GLP-1R^−/−^ with stable SNAP-GLP-1R wt or C438A expression, measured by DERET and expressed as fold increase from baseline, *n* = 5, “ns” indicates nonsignificant, by statistical tests indicated in the text. Data are shown as mean ± SEM, with individual replicates shown where relevant. Underlying raw data for all the panels included in this figure can be found in S2 Data; uncropped blots from this figure can be found in S1 Raw Images. DERET, diffusion-enhanced resonance energy transfer; FITC, fluorescein isothiocyanate; GLP-1R, glucagon-like peptide-1 receptor; HEK293; human embryonic kidney 293; HTRF, homogenous time-resolved fluorescence; TR-FRET, time-resolved Förster resonance energy transfer; wt, wild-type.
(EPS)

S5 Fig GIPR nanodomain clustering and palmitoylation. (A) Total input (“I”) and palmitoylated (“P”) SNAP-GIPR fractions from CHO-K1 cells stably expressing SNAP-GIPR and treated with vehicle (“Veh”) or 100 nM GIP for 10 min. (B) SNAP-GIPR distribution within TMFs, DSFs, and DRFs isolated from INS1 832/3 GIPR^−/−^ cells transiently expressing SNAP-GIPR after treatment with vehicle or 100 nM GIP for 5 min. (C) Comparison of SNAP-GLP-1R and SNAP-GIPR clustering, detected by HTRF, in transiently transfected HEK293 cells treated with vehicle, GLP-1 (100 nM), or GIP (100 nM) as indicated, *n* = 3; error bars have been omitted for clarity. Parallel measurement of receptor surface expression by Lumi4-Tb labeling and normalization to cell count also shown, paired *t* test. (D) Internalization of SNAP-GIPR in INS-1 823/3 cells expressing SNAP-GIPR and labeled for 30 min with SNAP-Surface 549 (red) followed by treatment with and without 10 mM MβCD for 1 h before stimulation with 100 nM GIP for 1 h. Nuclei (DAPI), blue; size bars, 10 μm, “ns” indicates nonsignificant. Data are mean ± SEM. Underlying raw data for all the panels included in this figure can be found in S2 Data; uncropped blots from this figure can be found in S1 Raw Images. CHO, Chinese hamster ovary; DRF, detergent-resistant fraction; DSF, detergent-soluble fraction; GIP, glucose-dependent insulinotropic polypeptide; GIPR, GIP receptor; GLP-1R, glucagon-like peptide-1 receptor; HTRF, homogenous time-resolved fluorescence; MβCD, methyl-β-cyclodextrin; TMF, total membrane fraction.
(EPS)

S6 Fig Biased agonist effects on GLP-1R nanodomain segregation and clustering—Extra data. (A) Kinetic traces of NR12S FRET in HEK293 SNAP-GLP-1R cells treated with the indicated agonist (100 nM), expressed as ratio of signals at 570 nm to 490 nm, representing Lo signal, or at 570 nm to 490 nm, representing Ld signal, and normalized to individual well baseline, *n* = 6, paired *t* tests comparing plateau determined from one-phase association fits. (B) Alternative analysis of data shown in (A), with AUC calculated from ratio of 570 and 610 signals, randomized block one-way ANOVA with Tukey’s test. (C) SNAP-GLP-1R internalization responses at indicated dose of each agonist in INS-1 832/3 GLP-1R^−/−^ SNAP-GLP-1R cells, measured by DERET and expressed as fold increase from baseline, *n* = 5. (D) Dose-response analysis of data shown in (C), with each agonist dose represented by AUC, 4-parameter logistic fit of pooled data shown. (E) SNAP-GLP-1R clustering responses at indicated dose of each agonist in INS-1 832/3 GLP-1R^−/−^ SNAP-GLP-1R cells, measured by HTRF and expressed as fold increase from baseline, *n* = 5. (F) Representative trajectories obtained during single-molecule SNAP-GLP-1R tracking, shown as MSD over time and classified into four different groups (immobile, subdiffusion, normal diffusion, and superdiffusion), based on their diffusion properties. *p < 0.05, **p < 0.01, “ns” indicates nonsignificant by statistical test indicated in the text. Data are shown as mean ± SEM. Underlying raw data used in S6F panel can be downloaded from: https://doi.org/10.6084/m9.figshare.c.4592000; underlying raw data for all other panels included in this figure can be found in S2 Data, and a dose-response summary for this figure is included in S7 Table. AUC, area under the curve; DERET, diffusion-enhanced resonance energy transfer; FRET, Förster resonance energy transfer; GLP-1R, glucagon-like peptide-1 receptor; HEK293, human embryonic kidney 293; HTRF, homogenous time-resolved fluorescence; Ld, liquid-disordered; Lo, liquid-ordered; MSD, mean squared displacement.
(EPS)

S7 Fig Biased agonist effects on GLP-1R nanodomain signaling—Extra data. (A) FRET traces with the indicated biosensors and agonists, normalized to individual well baseline, in CHO-SNAP-GLP-1R cells, *n* = 5 for each. (B) Dose-response curves constructed from data shown in (A) for ^T^Epac^VV^, AKAR4-NES and AKAR4-Lyn. For AKAR4-NES, signals have been combined into 6-min bins to improve precision and named according to the midpoint of each period, 3-parameter fits of pooled data shown. (C) TR-FRET kinetic binding data for exendin-4-K12-FITC and exendin-phe1-K12-FITC in INS-1 832/3 GLP-1R^−/−^ SNAP-GLP-1R cells with or without prior cholesterol depletion with MβCD (10 mM, 45 min), *n* = 7. (D) Association rate (*k*_on_), dissociation rate (*k*_off_), and equilibrium dissociation (K_d_) constants derived from (C), 2-way repeat-measures ANOVA with Sidak’s test comparing ± MβCD. *p < 0.05, “ns” indicates nonsignificant by statistical test indicated in the text. Data are shown as mean ± SEM, with individual replicates shown where relevant. Underlying raw data for all the panels included in this figure can be found in S2 Data, and a dose-response summary for this figure is included in S4 Table. CHO, Chinese hamster ovary; FITC, fluorescein isothiocyanate; FRET, Förster resonance energy transfer; GLP-1R, glucagon-like peptide-1 receptor; MβCD, methyl-β-cyclodextrin; TR-FRET, time-resolved FRET.
(EPS)

S8 Fig Effect of BETP on exendin-phe1 binding affinity and nanodomain signaling—Extra data. (A) TR-FRET kinetic binding data for exendin-4-K12-FITC and exendin-phe1-K12-FITC in INS-1 832/3 GLP-1R^−/−^ SNAP-GLP-1R cells cotreated with or without BETP (10 μM), *n* = 5. (B) Association (*k*_on_) and dissociation (*k*_off_) rate constants derived from (A), 2-way repeat-measures ANOVA with Sidak’s test comparing ± BETP. (C) Effect of BETP (10 μM) on exendin-phe1-induced β-arrestin-2 recruitment in PathHunter GLP-1R cells, 30-min incubation, 4-parameter fit of pooled data shown, *n* = 3. *p < 0.05, “ns” indicates nonsignificant by statistical test indicated in the text. Data are shown as mean ± SEM, with individual replicates shown where relevant. Underlying raw data for all the panels included in this figure can be found in S2 Data. BETP, 4-(3-benzyloxyphenyl)-2-ethylsulfinyl-6-(trifluoromethyl)pyrimidine; FITC, fluorescein isothiocyanate; GLP-1R, glucagon-like peptide-1 receptor; TR-FRET, time-resolved Förster resonance energy transfer.
(EPS)

S1 Table Dose-response summary for Fig 2 data. (XLSX)

S2 Table Dose-response summary for Fig 3 data. (XLSX)

S3 Table Dose-response summary for Fig 5 data. (XLSX)

S4 Table Dose-response summary for Fig 6 and S7 Fig data. (XLSX)

S5 Table Dose-response summary for Fig 7 data. (XLSX)

S6 Table Dose-response summary for S2 Fig data. (XLSX)

S7 Table Dose-response summary for S6 Fig data. (XLSX)

S1 Movie Time-lapse confocal microscopy of CHO SNAP-GLP-1R cells labeled with SNAP-Surface 549 for 30 min and treated with vehicle for 1 h before stimulation with 100 nM exendin-4. CHO, Chinese hamster ovary; GLP-1R, glucagon-like peptide-1 receptor.
(AVI)

S2 Movie Time-lapse confocal microscopy of CHO SNAP-GLP-1R cells labeled with SNAP-Surface 549 for 30 min and treated with 10 mM MβCD for 1 h before stimulation with 100 nM exendin-4. CHO, Chinese hamster ovary; GLP-1R, glucagon-like peptide-1 receptor; MβCD, methyl-β-cyclodextrin.
(AVI)

S3 Movie Time-lapse confocal microscopy of INS-1 832/3 GLP-1R^−/−^ cells stably expressing wild-type SNAP-GLP-1R following labeling with SNAP-Surface 549 for 30 min and stimulation with 100 nM exendin-4. GLP-1R, glucagon-like peptide-1 receptor.
(AVI)

S4 Movie Time-lapse confocal microscopy of INS-1 832/3 GLP-1R^−/−^ cells stably expressing C438A SNAP-GLP-1R following labeling with SNAP-Surface 549 for 30 min and stimulation with 100 nM exendin-4. GLP-1R, glucagon-like peptide-1 receptor.
(AVI)

S5 Movie Single-molecule TIRF videomicroscopy recorded in CHO-K1 cells transiently expressing SNAP-GLP-1R labeled with SNAP-Surface 549, vehicle conditions, including overlaid individual trajectories (blue). CHO, Chinese hamster ovary; GLP-1R, glucagon-like peptide-1 receptor; TIRF, total internal reflection fluorescence.
(M4V)

S6 Movie Single-molecule TIRF videomicroscopy recorded in CHO-K1 cells transiently expressing SNAP-GLP-1R labeled with SNAP-Surface 549, stimulated with 100 nM exendin-4, including overlaid individual trajectories (blue). CHO, Chinese hamster ovary; GLP-1R, glucagon-like peptide-1 receptor; TIRF, total internal reflection fluorescence.
(M4V)

S7 Movie Single-molecule TIRF videomicroscopy recorded in CHO-K1 cells transiently expressing SNAP-GLP-1R labeled with SNAP-Surface 549, stimulated with 100 nM exendin-phe1, including overlaid individual trajectories (blue). CHO, Chinese hamster ovary; GLP-1R, glucagon-like peptide-1 receptor; TIRF, total internal reflection fluorescence.
(M4V)

S8 Movie Single-molecule TIRF videomicroscopy recorded in CHO-K1 cells transiently expressing SNAP-GLP-1R labeled with SNAP-Surface 549, stimulated with 100 nM exendin-asp3, including overlaid individual trajectories (blue). CHO, Chinese hamster ovary; GLP-1R, glucagon-like peptide-1 receptor; TIRF, total internal reflection fluorescence.
(M4V)

S1 Raw Images Uncropped blots shown throughout the paper. (EPS)

S1 Data Excel spreadsheet containing, in separate sheets, the underlying numerical data for all panels in Figs 1–8. (XLSX)

S2 Data Excel spreadsheet containing, in separate sheets, the underlying numerical data for all panels in S1–S8 Figs. (XLSX)

## Abbreviations

2-BP: 2-bromopalmitate
AKAP: A-kinase anchoring protein
AP2: adaptor protein 2
AU: arbitrary unit
BETP: 4-(3-benzyloxyphenyl)-2-ethylsulfinyl-6-(trifluoromethyl)pyrimidine
cAMP: cyclic adenosine monophosphate
Cas9: CRISPR-associated 9
CCP: clathrin-coated pit
CHO: Chinese hamster ovary
CRISPR: clustered regularly interspaced short palindromic repeat
DERET: diffusion-enhanced resonance energy transfer
DRF: detergent-resistant fraction
DSF: detergent-soluble fraction
EGF: epidermal growth factor
EM: electron microscopy
FITC: fluorescein isothiocyanate
FRET: Förster resonance energy transfer
FSK: forskolin
GCGR: glucagon receptor
GIPR: glucose-dependent insulinotropic polypeptide receptor
GLP-1R: glucagon-like peptide-1 receptor
GPCR: G protein–coupled receptor
GαS: Gs alpha subunit
HA: hemagglutinin
HEK293: human embryonic kidney 293
IBMX: isobutylmethylxanthine
MSD: mean squared displacement
MβCD: methyl-β-cyclodextrin
PALM: photoactivatable localization microscopy
PAM: positive allosteric modulator
PK: ProLink
PKA: protein kinase A
siRNA: small interfering RNA
T2D: type 2 diabetes
TIRF: total internal reflection fluorescence
TM: transmembrane
TMR: 5-Carboxytetramethylrhodamine
TR-FRET: time-resolved FRET
YFP: yellow fluorescent protein
